# A large-scale field experiment across six rivers illustrates how the effects of resource enrichment are context dependent

**DOI:** 10.1007/s00442-023-05368-z

**Published:** 2023-05-03

**Authors:** William D. Bovill, Barbara J. Downes, Nick R. Bond, Paul Reich, Rhys Coleman, P. S. Lake

**Affiliations:** 1 School of Geography, Earth and Atmospheric Sciences, University of Melbourne, Parkville, VIC Australia; 2 Department of Infrastructure Engineering, University of Melbourne, Parkville, VIC Australia; 3 Centre for Freshwater Ecosystems, La Trobe University, Wodonga, VIC Australia; 4 Victorian Department of Energy, Environment and Climate Action, Melbourne, VIC Australia; 5 Melbourne Water, Docklands, VIC Australia; 6 School of Biological Sciences, Monash University, Clayton, VIC Australia

**Keywords:** Dispersal, Channel retentiveness, Species diversity, River invertebrates, South-eastern Australia

## Abstract

Resource supplementation can increase species richness and change the faunal composition of communities, but experiments have produced variable outcomes. An often overlooked element is that species richness can only increase if new taxa can disperse to resource-rich locations and invade established, local communities. We experimentally increased a basal resource (detritus) in six rivers in south-eastern Australia by driving wooden stakes into the riverbed to increase retention of detritus. Control sites were left untreated. Sites were located in agricultural sections with mostly cleared vegetation, but with intact (uncleared) reference sites upstream to provide sources of prospective colonists. We measured channel retentiveness and sampled benthic detritus and invertebrates before and after manipulation. We tested whether: greater retentiveness increased detritus densities, species richness and abundances and altered faunal composition; manipulation sites reached bio-equivalence with reference sites; new species arose from upstream reference areas; and whether outcomes were consistent across rivers. Only three rivers gained increases in detritus densities. All had low pre-existing amounts of in-stream wood compared with rivers that did not respond to treatment. Two rivers (Hughes Creek, Seven Creeks) gained higher species richness and invertebrate densities within 12 months and reached bio-equivalence with reference sites. In contrast, Turtons Creek showed species turnover through replacement of individuals. Only in Hughes Creek was there evidence of successful dispersal from the upstream reference area. The outcomes show that the effects of resource supplementation vary between rivers and suggest that pre-existing conditions (e.g. channel retentiveness) may cause these differences, providing clear evidence of context dependence.

**Supplementary Information:**

The online version contains supplementary material available at 10.1007/s00442-023-05368-z.

## Introduction

All ecological communities depend on basal resources (plants, detritus) that are exploited by primary consumers. Supplementation of these resources can produce marked changes in species diversity and community structure, especially over the short term (Yang et al. 2010). Nevertheless, outcomes of adding resources to communities can be complex, because both bottom-up and top-down effects may operate within food webs. Thus, an increase in basal food resources can lead to greater densities of primary consumers (e.g. Schneider et al. 2011) and greater species richness, potentially because rare taxa are able to attain densities that allow them to avoid local exclusion (Srivastava and Lawton 1998). However, spikes in species richness can subsequently collapse if resource-rich places ultimately attract predators that consume disproportionately higher numbers of prey (Holt 2008). Another complication is where natural resource supplementation happens in pulses at particular times of year. Resource pulses (such as leaf fall into streams) are characterised by high concentrations of resources over short periods of time relative to the timescale of consumers (Yang et al. 2010). Such pulses can create time lags in responses by consumers and their predators, further complicating the picture (Holt 2008).

A little-examined issue is that species richness can ultimately only increase with resource supplementation if new species are able to disperse successfully to resource-rich locations. Dispersal success requires not only that species have adequate dispersal ability to reach locations but are also able to invade existing local communities (Downes and Lancaster 2018; Brooks et al. 2020). “Invade” means the capacity of a species to enter a community and grow its population density; this follows terminology used in theories addressing species coexistence, which underpin questions about community assembly (Chesson 2008; HilleRisLambers et al. 2012). Dispersal success of prospective new species has not been a focus of much research, in part because many studies have been carried out over small spatial and temporal scales. Thus, when resources are supplemented in patches within localities, boosts to patch-level species richness come about by movement over short distances by individuals largely within the same community (e.g.: Dobson 1994), rather than by dispersal per se, i.e. by movement of individuals between separate, local communities. Additionally, studies conducted over short time scales may fail to identify density increases effected by new recruits following reproduction. Thus, tests are required that use multiple, natural, local communities and collect data over commensurately large scales of space and time.

In this study, we tested whether supplementing the basal resource of detritus would increase densities and diversities of invertebrates in rivers. Detritus that originates from terrestrial plants in riparian zones comprises leaves, bark and wood and can form the main energy source for riverine food webs (Reid et al. 2008) as well as provide many other resources such as shelter, oviposition sites, predator refuge, attachment points, etc. (Entrekin et al. 2008; Lancaster and Downes 2021). In general, detritus quality and quantity have strong effects on the diversity of organisms that depend on it (Gessner et al. 2010), but the effects of supplemented detritus on river ecosystems are unclear. Some experiments have demonstrated that supplementing detritus increased invertebrate densities and diversities (Smock et al. 1989; Richardson 1991; Dobson and Hildrew 1992; Wallace et al. 1997) but many studies have been carried out over relatively small spatial scales (such as replicate patches of detritus deployed within sites, e.g. Dobson 1994) or over larger scales but with limited replication (e.g. Wallace et al. 1999). An exception is an experiment carried out along 22 km of Hughes Ck (south-eastern Australia), which flows through an agricultural landscape where most terrestrial vegetation has been cleared. Detritus densities were boosted by hammering dozens of pairs of wooden stakes into the stream bed at 40 m-long experimental sites. The stakes improved retention by trapping drifting detritus on the stream bed, and comparisons with control sites revealed large increases in detrital densities, commensurate increases in species richness and densities of invertebrates, and marked changes in faunal composition (Lancaster and Downes 2017). Effects were sustained over 5 years, and detritus was shown to be a limiting resource for many taxa (Lancaster and Downes 2021). Nevertheless, the generality of this outcome remains untested; thus, it is unclear whether the strong effects of detritus supplementation in Hughes Ck can be expected in other rivers. Hughes Ck has a bed that comprises mostly sand, with very low, background standing stocks of detritus, and low invertebrate species diversity in areas without detritus (Downes et al. 2017). Hypothetically, characteristics such as these could affect the outcomes of detritus supplementation, such that rivers with coarser beds (e.g. cobble) or greater background standing stocks of detritus may exhibit different effects. Context dependence of this kind is a significant challenge for research that seeks to test hypotheses over large spatial scales (Catford et al. 2022) and has been encountered in previous work on resource subsidies (Subalusky and Post 2019). Knowledge of context dependence improves generality because it allows us to modify models to include important variables that act over large scales. In turn, this improves our ability to forecast correct outcomes for hypothesis tests when we have replicate ecosystems.

In this study, we replicated the Hughes Ck experiment in six rivers that flowed through agricultural landscapes; these included Hughes Ck but using a different reach of this river to that used in the original experiment. The six rivers are located within the same region of south-eastern Australia and span a variety of characteristics that could potentially affect experimental outcomes, thus providing a test for the generality of any response to experimental treatment. Streams were chosen using a formal process of selection from a statistical population and where candidate rivers had to meet a minimum set of criteria, some of which were necessary to implement the experiment. We make these criteria explicit and the procedure for choosing rivers transparent; this is a practice that is uncommon in studies of rivers (see Discussion) but is required to draw clear conclusions from empirical results. We posed the following questions:

1. Does increased retentiveness always increase densities of detritus at sites?
2. Does increased detritus increase species richness, densities of invertebrates and affect faunal composition?
3. Do manipulation sites attain detrital densities, species richness and numbers of invertebrate individuals that are comparable to intact, reference sites upstream?
4. Are new species sourced from upstream areas with intact riparian vegetation?
5. Are patterns consistent amongst all rivers, and between study periods, i.e. do all rivers show the same pattern or do results vary between rivers, and are results for Hughes Creek consistent with previous work?

At sites where densities of detritus are increased we predict that, if taxa are able to disperse to new locations and invade resource-rich sites successfully, then species richness should increase and faunal composition should change compared to control sites. Alternatively, if dispersal rates are low or dispersers are unable to invade established communities, then we expect no change in species richness or faunal composition. If there is no context dependence, then all six rivers should show similar outcomes. Alternatively, if one or more uncontrolled variables affect experimental outcomes, we expect results to vary between rivers. Our results and any context dependence have implications for stream restoration, because increasing channel retentiveness by adding stakes or other structures (e.g. large wood) to stream beds is a potential way to improve species diversity in degraded streams (Cornell et al. 2022) but may not work in all situations.

## Methods

### Experimental design

#### Selection of study rivers

To select rivers for the experiment, we followed a formal sampling process (Quinn and Keough 2002): i.e. we defined a statistical population of rivers, which included the criteria that rivers must meet to warrant selection, and outlined how candidate rivers were chosen. This was important, because sound conclusions about why ecosystems differ from one another require that replicate ecosystems are chosen using the normal requirements for sampling that enable statistical inference (Downes et al. 2002; Quinn and Keough 2002). Specifically: a statistical population must be defined, which will include essential criteria that ecosystems must fulfil to be included and usually a spatial boundary that demarcates the limits of the population (e.g. a region, a catchment, etc.); and, where there are more appropriate ecosystems than can be included in the study, selection should be done randomly. Transparent selection criteria are critical because they make explicit why some ecosystems were excluded from the sample; they also clarify the types of ecosystems to which the results can logically apply, regardless of whether all appropriate ecosystems were included (as is the case in this study) or were a random sample.

In this study, the initial statistical population was defined as all rivers falling within the Port Phillip, Goulburn Broken, North Central and West Gippsland basins of central Victoria, Australia. These basins were chosen because they had comparable climates, similar terrestrial vegetation and fell within a biogeographical region that would ensure aquatic species were relatively similar amongst rivers.

**Fig. 1:**
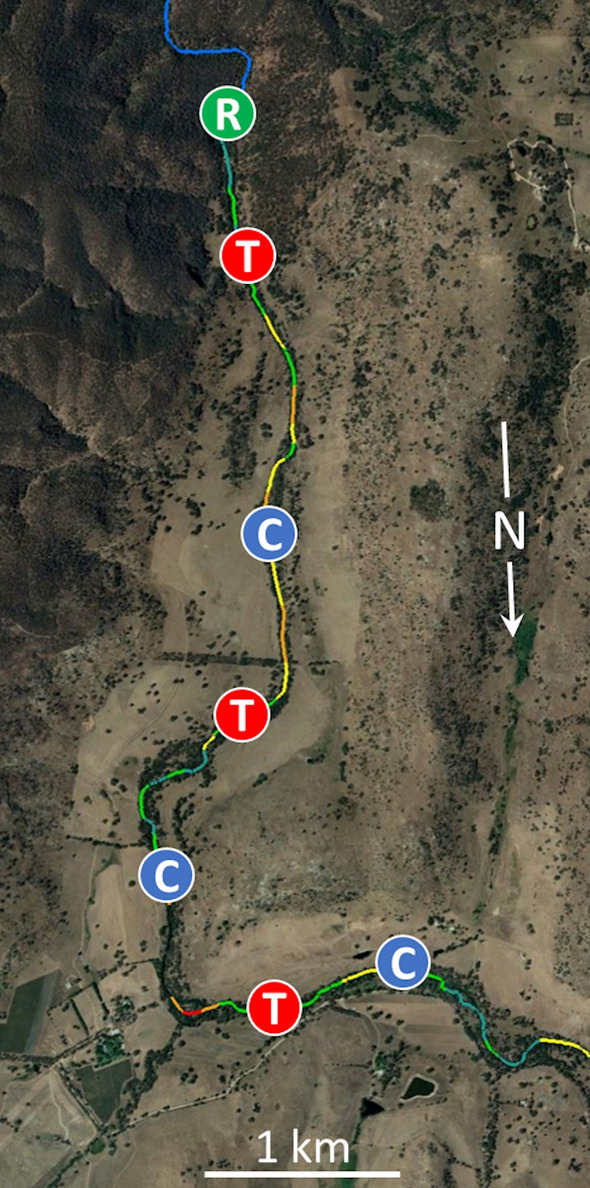
Example of a representative experimental reach (Hughes Creek). Experimental reaches included one reference site (R) in an upstream area with intact riparian vegetation; three control (C) and three treatment (T) sites distributed in agricultural landscapes with degraded riparian vegetation. Mean width of riparian vegetation, averaged across both banks, is colour-coded for contiguous 100 m sections of river channel (red < 10 m; orange 11–20 m; yellow 21–30 m; light green 31–40 m; dark green 41–50 m; blue > 50 m) (Victorian Department of Environment Land Water & Planning (DELWP) 2010)

**Fig. 2:**
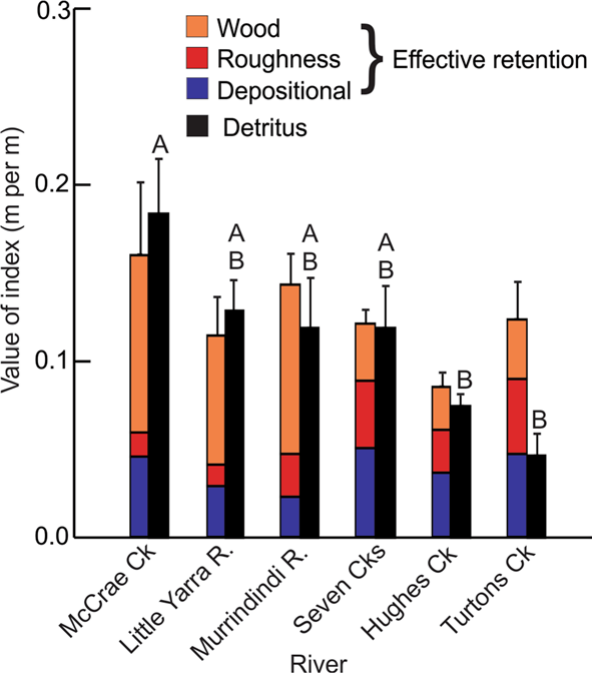
Estimates of the average densities (m/m) of effective retention structure (coloured bars) and detritus (black bars) across six sites in each of six creeks prior to experimental treatment (error bars are standard errors). Densities of detritus (m/m) that do not differ significantly from each other (using Tukey’s Honestly Significant Difference post hoc test) share a letter above the bar. Results were identical for standing stocks of detritus as measured by g.m.^−2^

Within these basins, rivers had to meet a set of criteria that were essential to permit the experiment to be implemented: (1) rivers must flow through agricultural landscapes that are cleared of forest, but have at least some riparian vegetation along channel sections where experimental sites are located (Fig. 1); (2) reaches upstream of the experimental section must have relatively high cover of riparian vegetation (e.g. average width of vegetation in riparian zone > 50 m, Fig. 1), i.e. a reference area that could provide a source of drifting detritus and be a prospective source of invertebrate colonists; (3) the channel should have no obvious severe human impacts (e.g. heavy stock access, weirs); (4) flow must be perennial and without poor water quality (e.g. not highly saline) and (5) overall experimental sections should be ~ 6–10 km long to ensure that all sites can be spaced approximately 1 km apart (Fig. 1). Previous work (Lancaster and Downes 2017—see their Supplementary Appendix S3) showed that a 1 km spacing is sufficiently far for sites to be statistically independent, but sufficiently close to allow dispersal between sites and from the source populations upstream.

Criteria 1–3 were assessed with a preliminary desktop exercise using vegetation and hydrological data from a state-wide assessment of river condition (Victorian Department of Environment Land Water and Planning 2010), which eliminated a large number of prospective rivers for use, largely because they did not meet the vegetation requirements. We followed up with an extensive scoping campaign involving site visits to a shortlist of 37 rivers during Spring 2016. Many of these potential rivers had low discharge after a relatively dry Spring, were already dry, or did not fulfil the criteria over a sufficient length of channel to accommodate our experimental design. Six rivers fulfilled all selection criteria (Little Yarra River, McCrae Creek, Murrindindi River, Seven Creeks, Turtons Creek and Hughes Creek, Supporting Information Fig. S1). Because we had the resources to replicate the experiment in six rivers, all six were selected (i.e. River is a fixed factor in our analysis). The six rivers ranged in mean channel wet width and composition of substrata, which varied from 100% sand to various mixes of clay, sand, gravel and cobbles (Supporting Information, Table S1).

#### Study sites

Seven sites were established in each river. All sites were 40 m long and established in sections of undivided channel without conspicuous bends, rocky outcrops, macrophyte beds, stock/vehicle crossings, etc. One site was situated upstream and located at random within a reference area (i.e. a location with relatively few human impacts (Downes et al. 2002)). Each reference site thus had relatively high cover of riparian vegetation, high detrital densities and potential for high species diversity of invertebrates as prospective colonists for sites downstream. We were unable to replicate reference sites within rivers due to practical constraints. Downstream of each reference site, six treatment sites (three control, three manipulation) were randomly located at ~ 1 km intervals along reaches flowing through cleared agricultural landscapes (Fig. 1). Treatments were assigned in a stratified random way by allocating treatments at random between the two upstream, two middle and two downstream sites. This process ensured control and manipulation sites were spread appropriately along the length of stream (Fig. 1).

Hughes Ck is unusual because, downstream of the agricultural area used for the previous experiment (Lancaster and Downes 2017), the river flows for ~ 5 km through a rocky gorge with intact vegetation before emerging into a second agricultural area. Thus, we were able to establish a new experimental length downstream of the original Hughes Ck experiment, because the rocky gorge fulfilled our requirement for an intact, upstream reference area.

#### Experimental manipulation

All sites were sampled for retentiveness, detritus densities and invertebrates prior to experimental treatment (between December 2016 and January 2017), and the manipulation was carried out immediately thereafter. We increased channel retentiveness at manipulation sites by hammering pairs of stakes vertically into the riverbed at a constant, average density of 0.4 stakes per m^2^ (Supporting Information, Figure S2) following Lancaster and Downes (2017). We used hardwood garden stakes (25 × 25 mm × 1.2 m) in sand beds and iron rebar stakes (12 mm × 1 m) in gravel/cobble beds to break through the hard substrata. Rebar stakes were capped for public safety (Danley™ LifeGuard™ Impalement Prevention Caps, https://www.danley.com.au). To expedite the retention response, initial supply was augmented by sourcing detritus from the banks and releasing it upstream of each site as per Lancaster and Downes (2017). Some detritus was retained and some drifted through the site. Thereafter, supply was not manipulated but determined by each river’s intrinsic supply and delivery of detritus to sites. The experiment ran for approximately 11 months prior to post-manipulation surveys in November 2017 (Supporting Information Table S2).

Mean daily flows during the experiment were sourced from Victorian Dept. of Environment, Land, Water and Planning (http://data.water.vic.gov.au/static.htm).

### Field sampling

Prior to experimental manipulation, the retentiveness of river channels and standing stocks of detritus were quantified along eight cross-sectional transects per site, using a modified line-intercept method (Bovill et al. 2020). We recorded the amount (m) of retentive elements intersected by each transect to calculate the average m of retention per m of transect (Linear Coverage Index: Brower and Zar 1977) as a measure of channel retentiveness. Ten types of retentive elements were surveyed, including log jams, wood, depositional zones, plants and cobbles (Bovill et al. 2020). We also quantified the amount (in m) of detritus (Coarse Particulate Organic Matter (CPOM), i.e. leaves, bark and wood ≤ 20 mm Ø) along each transect to quantify average standing stocks of detritus in m / m.

Before manipulation and at the end of the experiment, samples of invertebrates and detritus (CPOM) were collected from 15 random points within each site using a Surber net (30 × 33 cm frame; 250 µm mesh). The 15 samples were pooled (sites are replicates, not individual samples), and detritus and invertebrates were processed and enumerated following Lancaster and Downes (2017). Invertebrates were identified to the lowest taxonomic level possible, typically species or genus. Detritus was dried and weighed to determine g / m^2^.

### Statistical analyses

Retentiveness for each river was converted to ‘effective retentiveness’ following Bovill et al. (2020). Effective retentiveness weights the amount (m) of each retentive element by its Trapping Efficiency (i.e. the mean amount (m) of detritus retained by each m of that type of retentive element, averaged across sites). Background conditions of effective retentiveness, densities of detritus, channel characteristics and cover of terrestrial vegetation were compared between rivers (one way analysis of variance) and between sites allocated to different treatments (two-way analysis of variance).

For the experiment, analyses of variance were used to test for changes in detrital density, species richness and density of macroinvertebrates, as well as individual responses by common taxa. Analyses were run on log-transformed data (where necessary) using SYSTAT 12 (SYSTAT Software Inc.) with alpha set at 0.05. The model is a three-factor, repeated measures BACI-type design (Downes et al. 2002), with Treatment (fixed: control *vs* manipulation) crossed with River (fixed: six levels) and Site nested within the Treatment x River interaction term. River is a fixed factor because we used all rivers that met our selection criteria; these criteria therefore clarify the context of hypothesis tests, which is essential for evaluating mechanistic context dependency (Catford et al. 2022). The repeated measure of Time (before *vs* after) was crossed with both Treatment and River. A treatment effect is revealed by an interaction between Treatment and Time, and the manipulation sites following treatment should differ from all other treatment combinations. Therefore, we used the following a priori contrast to test for treatment effects for all variables:

$$\left({\overline{x}}_{BC}+{\overline{x}}_{BM}+{\overline{x}}_{AC}\right)-3.\left({\overline{x}}_{AM}\right)=0$$

where $\overline{x}$ and its subscripts indicate the mean of a relevant treatment combination, with *B* = before, *A* = after, *C* = controls and *M* = manipulation sites. Because our study rivers spanned a range of environmental conditions beyond our selection criteria (e.g. width and density of riparian vegetation, type of substrata, hydrological variation), we anticipated that the presence and strength of treatment effects would vary between them. Consequently, we carried out a test for treatment effects using the above a priori contrast for each river within the Treatment x Time x River interaction term. To preserve the independence of contrasts, the river with the smallest effect size was not tested; this was the Little Yarra R. in all cases (see below).

Effect sizes (ES) were calculated using the following formula:

$$ES=\frac{\left({\overline{x}}_{AM}-Y\right)}{Y}\times 100$$

where

$$Y=\frac{\left({\overline{x}}_{BC}+{\overline{x}}_{BM}+{\overline{x}}_{AC}\right)}{3}$$

Thus, when the manipulation created a significant treatment effect, the effect size was expressed as a percentage increase or decrease relative to controls. For tests on individual common species, 5% of significant outcomes may be chance events (i.e. Type I errors). However, we examine collective effects across communities, which do not depend on the outcomes of tests for specific species.

Where we observed significant increases in detrital densities, species richness or invertebrate densities, we wished to test the hypothesis that mean values at manipulation sites were similar to those seen at reference sites. A successful outcome would mean that we expect no differences, but this hypothesis has no natural null (because it is itself a null hypothesis), and this creates serious problems with inference (see Underwood 1990 for a full discussion). To overcome this problem, we used bio-equivalence (McDonald and Erickson 1994). In this approach, we calculate the ratio, *R*, of the mean of manipulation sites to that of a target (e.g. reference) condition, and test an hypothesis that *R* ≤ *R*_*l*_, where *R*_*l*_ is a predetermined, deemed lower limit of minimum equivalence (e.g. 0.8). Rejection of this one-tailed hypothesis leads to acceptance of the alternative hypothesis that *R* > *R*_*l*_. Additionally, confidence intervals around estimates of *R* reveal whether values include 1.0 (i.e. full equivalence: see Downes et al. 2002 for examples). To create target values for hypothesis tests for each relevant variable, we calculated averages across the six reference sites. Given each site is randomly located on a river that met a common set of environmental criteria, we argue that these six values adequately represent the reference condition for rivers in our population. We used *R*_*l*_ = 0.8 for hypothesis tests.

The same three-factor, repeated measures model given above was used to run PERMANOVA in the programme PRIMER 6 (PRIMER-E Pty Ltd) to examine changes in faunal composition. Abundances were 4th root-transformed and then converted to a similarity matrix using the Bray–Curtis similarity coefficient (Anderson et al. 2008). Significance levels of model terms were evaluated using 9999 permutations. Differences in faunal composition were displayed using non-metric multidimensional scaling (NMDS) solved in two dimensions, and SIMPER was used to identify which taxa contributed most to treatment differences (Clarke and Gorley 2006).

We also carried out NMDS that included reference sites to examine whether changes in faunal composition at manipulation sites converged on those seen at the reference sites. The similarity between each manipulation site and the reference site following manipulation was averaged and divided by the comparable, average similarity seen amongst control sites. We again used a bio-equivalence approach by testing a one-tailed hypothesis that the value of this ratio is ≤ 1. If this hypothesis is accepted, then the manipulation sites are no more similar to the reference site than controls. If the hypothesis is rejected, then we accept the alternative hypothesis that the manipulation sites are significantly more similar to the reference site than are the controls.

## Results

### Differences between study rivers

Prior to the start of the experiment, total effective retentiveness did not vary between rivers (*P* > 0.05), but amounts of woody retentive structure were significantly higher in three of the six rivers (*P* = 0.01) (Fig. 2). Additionally, detrital standing stocks were significantly higher (more than twofold) in McCrae Ck than in Hughes and Turtons creeks, with the other creeks having intermediate amounts (Fig. 2). Within each river, control and manipulation sites did not vary in total effective retention or standing stocks of detritus at the start of the experiment (as measured by metres of transect intercepted by detritus and by g.m^−2^; *P* > 0.05 in both cases).

**Fig. 3:**
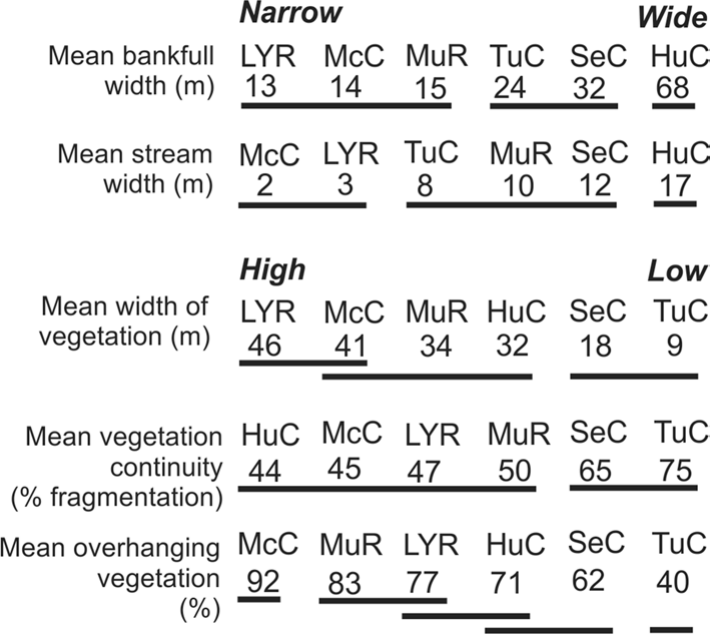
Differences between the experimental sections of six study rivers, ranked by channel width (*Narrow–Wide*) and vegetation cover in the riparian zone (*High–Low*). LYR, Little Yarra R.; McC, McCrae Ck; MuR, Murrindindi R.; HuC, Hughes Ck; SeC, Seven Cks; TuC, Turtons Ck. Numbers below acronyms provide actual mean values. Values connected by a horizontal line are not significantly different (see Fig. S3 for further information)

Little Yarra River and McCrae and Turtons creeks had narrow channels compared to Hughes Creek, which was almost five times wider, both in terms of bankfull widths and stream bed widths (Fig. 3, Supporting Information, Fig. S3). McCrae Ck and the Little Yarra R, along with Murrundindi R, had the highest overall cover of terrestrial vegetation across multiple measures. In contrast, both Seven and Turtons creeks had narrow riparian zones, highly fragmented vegetation and little overhanging vegetation (Fig. 3). The reference site on each creek occurred within areas having typically much higher cover of vegetation than the experimental sections (Supporting Information Fig. S3).

Hydrographs revealed that rivers varied in relative stage heights (i.e. depths) during the experimental period (Supporting Information Fig. S4). McCrae and Turtons creeks and Little Yarra R. attained flows that were ~ 95%, 73% and 65%, respectively, of bankfull, whereas the other rivers had relatively low peak flows.

### Experimental effects on detritus and invertebrates

**Fig. 4:**
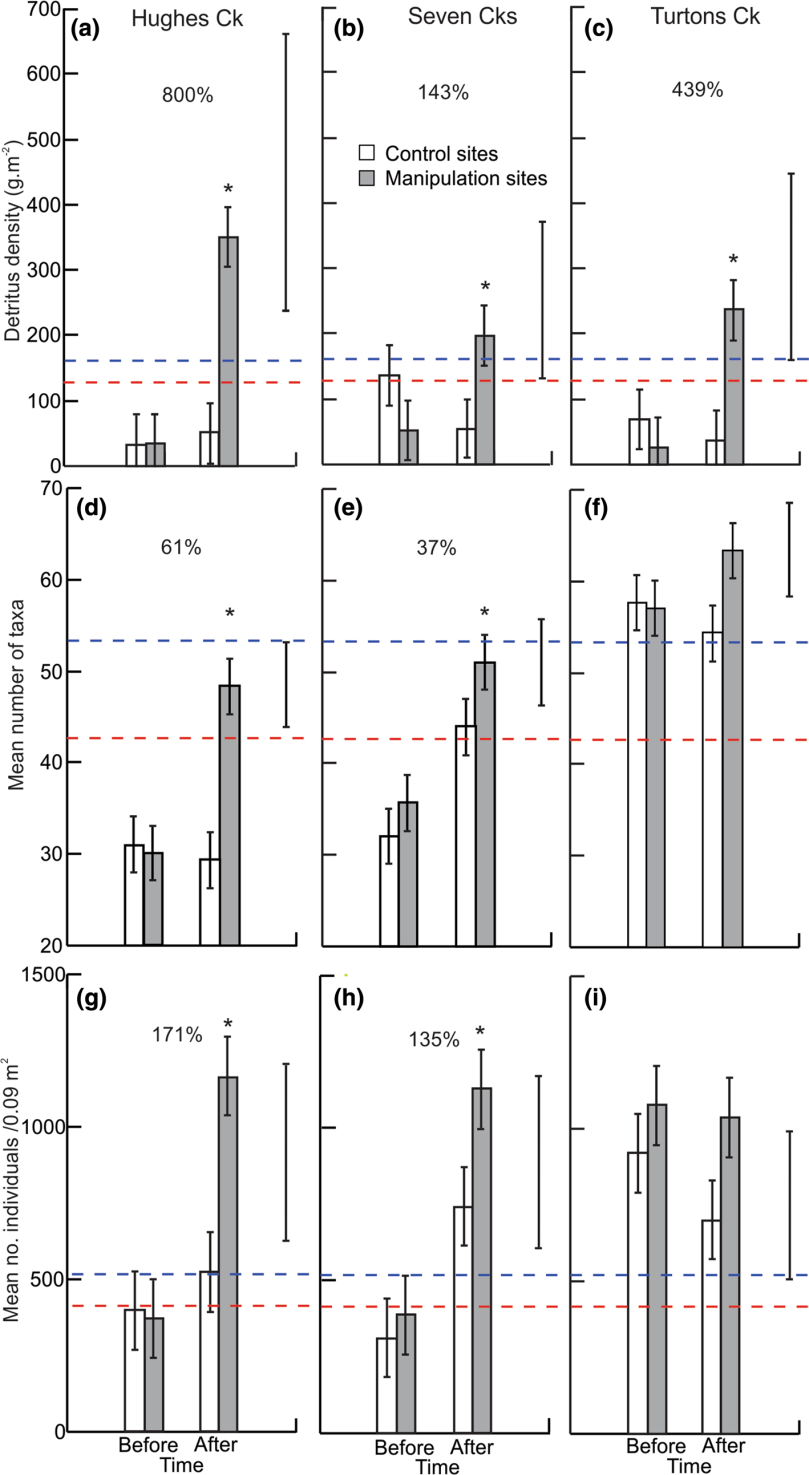
Average standing stocks of detritus (**a**–**c**), numbers of taxa (**d**–**f**) and invertebrate densities (**g**–**i**) at control (*n* = 3 per river) and manipulation (*n* = 3 per river) sites plotted both before and after manipulation for each of three rivers (in columns) that showed significant responses. Asterisks above bars indicate that manipulation sites following treatment were significantly different to controls (*P* < 0.05; see results of contrasts in Table 1). Percentages in each panel give effect sizes. Error bars are standard errors derived from the appropriate MS term from analyses (which in all cases was MS_residual_). Also shown are 0.8 bio-equivalence values to reference sites (red dotted line) and 1.0 equivalence values (i.e. means of reference sites) (blue dotted lines) as well as the 90% confidence interval around each estimate of *R* (solid black line on right hand side of each graph). The hypothesis that *R* ≤ *R*_*l*_ is rejected if the lower confidence interval does not intersect with a bio-equivalence of 0.8 (i.e. the red dotted line), which supports the hypothesis that *R* > *R*_*l*_

Densities of detritus increased strongly at manipulation sites in Hughes, Seven and Turtons creeks (Table 1, Fig. 4), but not in the other three rivers (Table 1, Supporting Information Fig. S5), even though stakes accumulated large amounts of detritus at all sites (Supporting Information Fig. S2). In rivers showing a significant treatment effect, densities of detritus either matched (Seven Cks) or exceeded (Hughes Ck, Turtons Ck) the densities of detritus at upstream reference sites (Fig. 4) and also either matched or exceeded typical detrital densities in the other three rivers (Murrindindi R., McCrae Ck, Little Yarra R.: Fig. S5).

**Table 1:** Results from repeated measures ANOVA comparing density of benthic detritus, taxon richness and densities of individuals in different treatments (manipulation sites vs controls) in six rivers and over time (before *vs* after), plus a priori contrasts (see Statistical analyses) for each river (indented)

		CPOM density (g.m^−2^)			Taxon richness			Density of individuals		
Source	df	MS	*F*	*P*	MS	F	P	MS	*F*	*P*
Treatment	1	98,901	8.60	**0.007**	196.1	2.68	0.11	0.14	3.85	0.06
River	5	39,027	3.39	**0.019**	920.6	12.53	** < 0.001**	0.25	7.08	** < 0.001**
Treatment x River	5	15,288	1.33	0.28	54.8	0.75	0.60	0.02	0.61	0.69
Between sites (within Treatment x River)	24	11,500			73.5			0.04		
Time	1	58,138	13.90	**0.001**	506.7	23.50	** < 0.001**	0.23	9.50	**0.005**
Time x Treatment	1	175,143	15.39	** < 0.001**	253.1	10.12	**0.004**	0.07	3.04	0.09
Time x River	5	17,615	1.55	0.21	134.4	6.76	** < 0.001**	0.14	5.55	**0.002**
Time x Treatment x River	5	20,687	1.82	0.14	39.7	2.82	**0.039**	0.03	1.08	0.40
Hughes Ck	1	397,909	34.60	** < 0.001**	747.11	28.42	** < 0.001**	0.44	17.79	** < 0.001**
Seven Cks	1	54,686	4.76	**0.039**	427.1	16.25	** < 0.001**	0.44	17.74	** < 0.001**
Turtons Ck	1	152,217	13.24	**0.001**	110.3	4.19	**0.05**	0.0001	0.004	0.95
Murrindindi R	1	10,736	0.93	0.34	205.4	7.81	**0.01**	0.01	0.51	0.48
McCrae Ck	1	29,906	2.60	0.12	4.69	0.18	0.68	0.07	2.93	0.10
Residual error	24	11,378			26.29			0.03		

The river with the smallest effect size was not tested (to maintain independence of contrasts), which was the Little Yarra R. in all cases. df, degrees of freedom; MS, mean squares; *F* value of F ratio, *P* probability. *P* values in bold are equal to or less than 0.05

Taxon richness of invertebrates increased significantly in Hughes Creek and Seven Creeks and was just marginally non-significant in Turtons Ck (Table 1, Fig. 4). The particularly large increase in Hughes Ck saw its relatively low taxon richness raised to numbers comparable with reference sites and other rivers, including those that did not respond to treatment (Fig. S5). There was also a small increase in taxon richness in the Murrindindi River (Table 1; Supporting Information Fig. S5). Overall densities of invertebrates increased at Hughes and Seven creeks, but not in Turtons Creek (Table 1, Fig. 4). Final invertebrate densities in the manipulation sites of Hughes and Seven creeks were double those seen in the three rivers that did not respond to treatment (Supporting Information Fig. S5) and matched values seen in reference sites (Fig. 4).

**Fig. 5:**
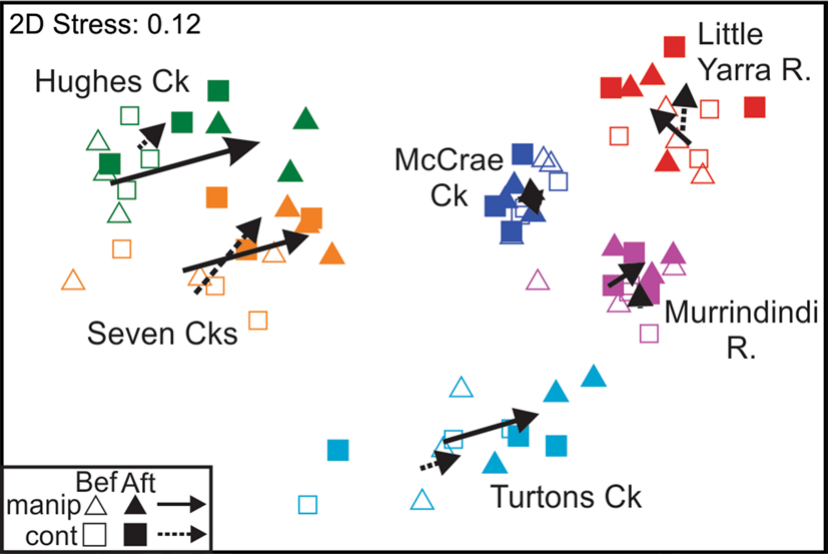
Non-metric multidimensional scaling (NMDS) plot showing manipulation (*n* = 3) and control (*n* = 3) sites in each creek, before and after the experimental manipulation. The centroid of the before values (arrow start) to after values (arrow end) indicate the direction and extent of change for control (dashed line) and manipulation (solid line) sites

**Fig. 6:**
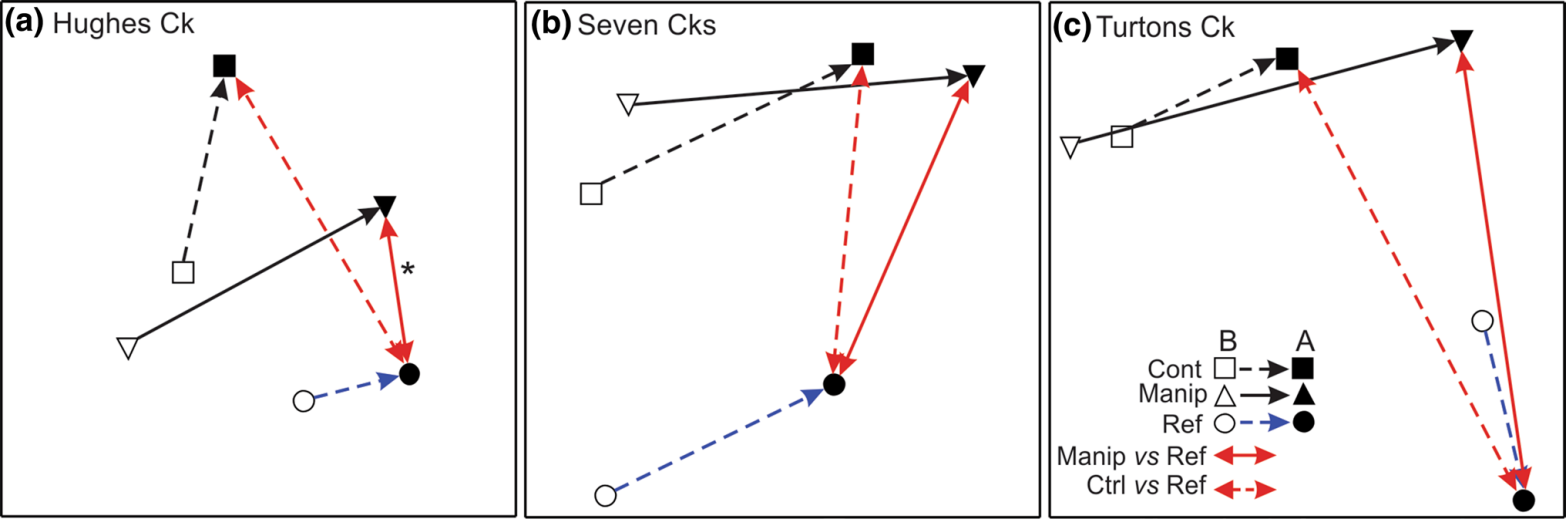
Non-metric multidimensional scaling (NMDS) plots showing changes in faunal composition for each of three creeks (**a**–**c**) that showed treatment effects. Black arrows connect the centroid of control (dashed) or manipulation (solid) sites from before (B) to after (A) experimental manipulation; blue arrows connect individual reference points from before to after. Red, double-headed arrows illustrate the differences in similarity between either control (dashed lines) or manipulation (solid lines) sites and the reference site after manipulation. An asterisk indicates treatment sites are significantly more similar to the reference site than controls (see Table S3). The NMDS was carried out on all data (as per Fig. 5) but rivers have been plotted separately for clarity. The ranges of values on axes are different for each plot, which should not, therefore, be compared directly

Faunal composition differed significantly between manipulation and control sites after treatment (Time x Treatment term, Table 2). Pairwise tests suggest that control and manipulation sites were similar prior to treatment (*t* = 0.90, *P* = 0.73) but were significantly dissimilar following treatment (*t* = 1.58, *P* = 0.002). Although the three-way interaction (Time x River x Treatment) was non-significant (Table 2), scrutiny of the average dissimilarities between control and manipulation sites suggest that the Time x Treatment interaction was primarily driven by outcomes in Hughes and Turtons creeks, which both showed a strong increase in dissimilarity between control and manipulation sites following treatment, whereas other creeks did not (Supporting Information, Fig. S6). An NMDS plot shows that the faunal composition of manipulation sites diverged away from the controls in Hughes and Turtons creeks, and converged on those of the three rivers that had highest initial standing stocks of detritus (Fig. 5). These latter three rivers (McCrae Ck, Little Yarra R. and Murrindindi R.) exhibited only small and variable changes in composition that probably reflect seasonal effects (Fig. 5). In Seven Creeks, the direction and size of change in the manipulation sites were similar to both Hughes and Turtons creeks, but the controls showed a similar size and direction of change (Fig. 5), such that similarity amongst all six sites increased following the manipulation (Supporting Information, Fig. S6). NMDS plots that included the reference sites and the averages of the experimental and control sites showed that Hughes Ck was the only river to show a significant increase in similarity between the manipulation sites and its reference site compared to controls, following treatment (Fig. 6). The size of increase in similarity was comparable to that seen in the original Hughes Ck experiment (Table S3).

**Table 2:** Outcomes of analysis of experimental effects on faunal composition across six rivers using PERMANOVA

Source	df	SS	MS	Pseudo-F	P(perm)
River	5	57,787	11,557	16.031	**0.001**
Treatment	1	774	774	1.074	0.36
River x Treatment	5	3055	611	0.848	0.85
Between sites within (River x Treatment)	24	17,303	721		
Time	1	3702	3702	8.167	**0.001**
Time x Treatment	1	1168	1168	2.578	**0.003**
Time x River	5	6614	1323	2.9198	**0.001**
Time x River x Treatment	5	2326	465	1.026	0.41
Residual	24	10,878	453		

Pseudo-F, pseudo-F values; P(perm), probability values gained by permutation test; for other terms see Table 1

* P* values in bold are less than 0.05

SIMPER analyses (Supporting Information, Tables S4, S5) showed that treatment effects on faunal composition were delivered by multiple taxa that each contributed a small percentage. In both Hughes and Turtons creeks, the top contributors to faunal differences were spread over a wide variety of taxa: beetles, stoneflies, dipterans (blackflies, chironomids) and caddisflies (hydropsychids and some cased caddisflies); they also often featured early instars. Many of these taxa were collectively abundant in the three creeks with the highest standing stocks of detritus: McCrae Ck and Murrindindi and Little Yarra rivers (Supporting Information, Tables S4, S5). Hughes Ck was characterised by strong differences between manipulation and control sites with high average dissimilarity compared to standard deviation, which indicates relatively consistent effects across sites (Supporting Information, Table S4). In contrast, Turtons Creek had smaller differences between manipulation sites and controls, few taxa absent from controls, and many taxa with lower abundances at manipulation sites (Supporting Information, Table S5).

### Experimental effects on common taxa

Sixty-five, one-hundred and sixty-eight and taxa in Hughes, Turtons and Seven creeks, respectively, were sufficiently abundant to permit analyses of their individual responses. Approximately half of these common taxa responded to treatment (“responders”) in Hughes Ck, which is consistent with the previous experiment on Hughes Ck (Lancaster and Downes 2017), whereas only about one-third responded in Turtons and Seven creeks (Table 3, Supporting Information Tables S6-S8). Overall, proportions of responding species in each river were much higher than expected by chance (5%, due to Type I errors). Most responders attained greater densities at manipulation sites, with effect sizes ranging from 20% to > 1000% (i.e. an 11-fold increase). Some common taxa appeared at manipulation sites only after treatment (e.g. several stoneflies and beetles), with Hughes Ck having the highest number of such new colonists. A few common species declined in densities at, or were lost from, manipulation sites, particularly in Turtons Ck. Notably, some of these were grazing taxa (e.g. the beetle *Sclerocyphon* sp. and the cased caddisfly *Agapetus* sp.).

**Table 3:** For each of three rivers, the number of common taxa (defined as being sufficiently abundant to permit the analysis) that responded significantly to treatment (“responders”, with either increased or decreased densities) and the number that did not respond to treatment out of the total

Outcome	Hughes Ck	Turtons Ck	Seven Cks
Positive treatment effect			
No. of taxa with increased densities	21	19	19
Number of new taxa	11	4	3
Total	**32**	**23**	**22**
Range of effect sizes	21–1011%	20–965%	21–1122%
Negative treatment effect			
No. of taxa with decreased densities	2	8	0
Number of taxa lost	1	5	3
Total	**3**	**13**	**3**
Range of effect sizes	-34 – -50%	-14 – -73%	
No response	30	64	43
TOTAL	**65**	**100**	**68**

For responders, reported are numbers of species with changed densities, along with the range in effect sizes, and the number of new taxa or taxa that were lost at manipulation sites

Values in bold are sub-totals or total numbers of taxa

Responders were spread across a wide array of types of species in all three creeks and notably included many early instars (Supporting Information Tables S6-S8). Thirty-one taxa were common to all three creeks but only nine produced consistent results across all creeks. Of those that responded positively to treatment, two were stoneflies (*Dinotoperla thwaitesi*, *Illiesoperla australis*), one a chironomid (*Harrisius* sp.), two were beetles (*Notriolus maculatus* larvae, *Notriolus* spp. early instar larvae) and one was a caddisfly (*Cheumatopsyche* spp. early instars). All of these taxa except *Cheumatopsyche* consume detritus directly (Chessman 1986). We also compared the list of responders and non-responders in Hughes Creek to results from the earlier experiment. Of 53 common taxa in the earlier experiment, 31 were recorded in the current study and 21 of these had consistent responses in both experiments. Of the ten remaining taxa, six responded only in the earlier experiment and four responded only in the current experiment (Supporting Information, Table S6).

## Discussion

We tested whether improved retentiveness would increase standing stocks of benthic detritus and, consequently, species diversity and densities of macroinvertebrates in six rivers that varied in channel morphology, substrate size and density of surrounding terrestrial vegetation. We found that increased retentiveness boosted detritus densities in only half of our rivers, but those rivers showed greater species richness or species turnover and changes in faunal composition. Increased densities of detritus enabled new taxa to invade established, local communities; this suggests that individual taxa frequently disperse along rivers but are unable to invade sites when resources are meagre or fully monopolised by resident taxa. These outcomes are broadly consistent with the previous experiment in Hughes Creek, but differences between rivers in whether and how effects were manifest showed that resource supplementation has context dependence.

Standing stocks of detritus increased in only three of the six rivers, and this variable outcome broadly reflects conflicting results in the literature (Lepori et al. 2005; Entrekin et al. 2008; Lancaster and Downes 2017). A potential explanation for such differences is that the amount, type and effectiveness of pre-existing retention play a strong role. Notably, the three rivers where detritus did not increase had large proportions (65–69%) of existing retention created by wood, which is highly effective at retaining detritus (Webster et al. 1994; Bovill et al. 2020) even during periods of high discharge (Jones Jr and Smock 1991). The simplest explanation is that existing retention in these three rivers was adequate to retain all drifting detritus. Thus, whilst stakes accumulated large amounts of detritus (Supporting Information Fig. S2), they trapped material that would have been otherwise retained by existing wood, leading to no net increase. If that is correct, then standing stocks of detritus in these streams are determined by the supply of material from the riparian zone, not retentiveness.

In contrast, the three rivers showing increased detritus had lower background densities of wood-based retention (28–30%) and much higher proportions of retention created by low flows in depositional areas (35–40%, compared to 14–25% in rivers without a treatment effect). Depositional areas trap detritus efficiently but they often disappear at high discharges, releasing detritus downstream (Jones Jr and Smock 1991). It seems likely that depositional areas in this experiment were flushed of detritus during peak discharges in late winter and spring (Supporting Information Fig. S4); these areas would not have been replenished prior to final sampling (late spring/early summer), because peak leaf and bark fall occur in late summer (Lake 1995; Downes et al. 2017). Consequently, control sites in these three rivers would have lost much of their detritus during winter. In contrast, the manipulation sites gained high detritus densities because wooden stakes retain detritus throughout the year (Lancaster and Downes 2017). Critically, benthic detrital densities in these three rivers were raised to levels that were comparable to (or even exceeded) those seen in study rivers that have greater cover of terrestrial vegetation. This outcome suggests that it was the lack of effective retentive structure that limited detrital densities in these rivers rather than low supplies of detritus from surrounding terrestrial vegetation.

Resource enrichment enabled new species to invade treatment sites and raised species richness markedly in two rivers (Hughes Ck, Seven Cks) and with a marginally non-significant outcome (*P* = 0.05) in the third (Turtons Ck). These results are consistent with a scenario where additional resources enable new species that disperse routinely through sites to establish themselves in the benthos, rather than extra resources simply bolstering the density of species already present. Importantly, the outcome shows that new species were able to invade an existing local community, and this suggests that priority effects via niche pre-emption were not strongly at play. That outcome matches other, similar, field-based experimental studies in rivers (Brooks et al. 2020; Lancaster and Downes 2021) but contrasts with much of the literature. The latter suggests that priority effects can prevent invasion by new taxa (Fukami 2015), but most of those studies (except those on plants) are based on laboratory or mesocosm research. There are very few studies that have examined community invasibility over appropriately large spatial and temporal scales, in field settings with existing local communities, and where the species identity and frequency of dispersers are naturally determined.

Turtons Creek differed from Hughes and Seven creeks in that invasion by new species created compositional changes and species turnover rather than clear increases in species richness. Overall invertebrate densities did not increase in Turtons Ck, and a relatively large number of species decreased in abundance or were lost outright from manipulation sites. Thus, the compositional changes in Turtons Ck came about through replacement of some individuals of existing species, rather than by addition. In contrast, both Hughes and Seven creeks gained higher densities of individuals, and thus new species were accommodated by addition rather than replacement. Both Hughes and Seven creeks are sandy bed streams with few hard surfaces (cobbles, wood). Increases in detritus added new resources related to the provision of hard surfaces and physical complexity that is created by detritus. In contrast, Turtons Ck is a predominantly gravel-bed stream, and therefore has relatively ample resources of space that are supplied by hard surfaces and existing physical complexity. It is unclear why addition of detritus would necessarily result in species turnover, but type of substrata potentially provides a different context for the hypothesis test.

Increased detritus caused the faunal composition of both Turtons and Hughes creeks (and, to some extent, Seven Cks) to become more similar to rivers having high natural densities of detritus. Nevertheless, only a few individual taxa consistently responded to increased detritus. Most repeat responders shredded leaves or consumed wood, and this outcome is consistent with effects of detritus on invertebrate taxa elsewhere (Lepori et al. 2005). However, there is a paucity of obligate shredders in agricultural rivers in this region (Reid et al. 2013), which may explain why so few taxa responded consistently. Possibly, some responses to detritus are dietary related but indirect (e.g. predators that respond gradually to increased numbers of prey accumulating within leaf packs) and take time to appear. However, many taxa exploit detritus for other, essential resources that can be otherwise limiting (Lancaster and Downes 2021), such as hard surfaces (attachment for filter-feeders, biofilm for grazers) or physically complex living spaces that provide hydraulic or predator refuges. Nonetheless, one consistent pattern witnessed in this study was that many responders were early instars. Some stream invertebrates lay eggs on wood or bark, and females will increase egglaying in response to increased detritus (Macqueen and Downes 2015). Thus, detrital packs can also enhance recruitment by increasing sites for oviposition and may also provide nurseries for hatchlings. For example, early instars of some caddisflies use particular types of detritus to build their first cases (Otto and Svensson 1980), which shows detritus is used in a variety of ways. Similarly, Entrekin et al. (2008) found higher densities of the shredder *Lepidostoma* (a cased caddis) 2 years after wood was added. *Lepidostoma* uses wood for pupation, and wood additions may have increased the local supply of adults, thus eventually enhancing recruitment. Such observations suggest that effects of increased detritus on life cycle stages other than larvae must be examined.

Unlike Hughes Ck, there was no evidence that upstream reference areas supplied the new taxa that established themselves in treatment sites in Seven and Turtons creeks, even though distances between reference areas and experimental lengths were similar to that of Hughes Ck and many common taxa were also the same. Instead, new species may have arrived by drifting over short distances within the overall experimental length. This movement can nevertheless boost overall population densities of these taxa if detritus increases survivorship. In contrast, manipulation sites in Hughes Ck became more similar to reference sites, and thus gained taxon richness via new species originating from upstream locations; this was also the predominant source of colonists in the original experiment on a different length of Hughes Ck (Downes and Lancaster 2018). The outcomes of the two experiments are thus consistent and suggest that Hughes Ck has particular characteristics that facilitate a strong response in species diversity from upstream species pools.

A big challenge in ecology is to discover how or whether large-scale factors override or interact with experimental variables to change outcomes of tests in different places (mechanistic context dependence: Catford et al. 2022). Multiple forms of mechanistic context dependence were evident in our experiment: increased retention boosted detritus densities only in those streams lacking effective retentiveness, regardless of detrital supply; higher detrital densities either increased species richness or created species turnover, with this difference possibly related to the type of benthic substrata; and upstream reference areas supplied new colonists to treatment sites in one stream (Hughes Ck) but not others. Hughes Ck also had overall the strongest responses to increases in detritus densities, and its consistent responses across two experiments suggest that it has particular characteristics—possibly related to its sandy bed and very low background densities of detritus—that deliver strong responses to increases in detritus. However, the results also show that Hughes Ck is exceptional; it did not ultimately provide accurate guidance for expected outcomes in other streams, even the one most similar to it (Seven Cks), which underlines the importance of replicating experiments across different ecosystems.

### Placing our results into context: where does generality come from?

Arguably, the broadest generality is delivered by meta-analyses of multiple studies from different parts of the world; this is because individual studies are usually spatially limited, and results may vary considerably between ecosystems in different regions, continents or hemispheres. When arguing the generality of individual studies, however, a frequently overlooked, essential aspect is the method by which replicate ecosystems are selected. We used a transparent process to select the rivers used in this study, by identifying a statistical population of rivers and selecting appropriately those rivers that met a priori criteria. By following a formal sampling protocol, we are permitted to draw conclusions about rivers of this kind in our population, and to propose that our findings may apply to rivers meeting the same criteria elsewhere. Tests of such hypotheses are where generality can come from in empirical research.

It is uncommon, however, for river researchers to follow formal sampling protocols to select rivers. In a brief survey of 50 published studies that report results from hypothesis tests across multiple rivers, only 3 (6%) clarified the statistical population from which rivers were drawn, and only 2 described criteria that allowed rivers to qualify for inclusion and an appropriate sampling process for selection (Supporting Information, Appendix S1, Table S8). Nevertheless, some studies we reviewed drew firm interpretations about the causes of between-river variation yet provided no rationale for the choice of rivers. Other studies chose individual rivers and, without explanation, deemed them to be “representative” of some type. In our study, disparities between Hughes Ck and other streams meeting the same selection criteria illustrate the dangers of assuming that a stream of a particular “type” can represent others of that type without evidence. The quality of research and ultimately of meta-analyses based on such research would be greatly improved if the process of selecting ecosystems for study was transparent and met the requirements for sound inference.

### Management implications

We have shown that boosting detritus densities in rivers that flow through agricultural landscapes and that lack much in-stream wood can increase the species richness and abundances of invertebrates and bring their invertebrates into bio-equivalence with reference locations that have intact vegetation. Furthermore, a separate study in Hughes Ck using exactly the same experimental protocols showed increased detritus boosted the abundances of small-bodied species of fish, some of which are threatened or endangered (Cornell et al. 2022). These outcomes suggest that wooden stakes could be used to improve species diversity in rivers that have been degraded by the removal of in-stream wood.

Replanting riparian revegetation is probably the most cost-effective long-term strategy for broad-scale restoration (Hale et al. 2018) but can take decades to deliver significant amounts of fallen wood to channels. At places where detritus is limiting, managers should therefore expect invertebrates to respond over similar timeframes. The process may be hastened by planting fast-growing, short-lived (~ 10 years) riparian species (e.g. in Australia, some species of *Acacia* and *Leptospermum*) that contribute large wood more quickly than long-lived species. In streams lacking structural retentiveness, it might be desirable to fast-track the response even further by adding wooden stakes, which can increase benthic detritus and invertebrate densities quickly. Success of the method requires that streams have inputs of detritus from local or upstream riparian vegetation, but large increases in detritus stocks were observed even in streams with relatively poor riparian vegetation. Strongest biological responses may be seen in sites where local abundance and diversity of invertebrates is poor, but where source populations of prospective colonist species are present in less degraded areas upstream. Stakes are particularly effective because they create variable sizes and composition of detritus packs, which support different invertebrate species (Lester et al. 2007). In contrast, adding large logs creates debris dams that are required by some species (e.g. large-bodied fish: Roni et al. 2008) but can be unsuitable for many taxa that prefer multiple, small detritus packs (Cornell et al. 2022).

Wooden stakes are cheap and are quickly deployed at multiple locations without specialised skills or equipment. They are durable in places without strong erosion or deposition of sediments and functioned effectively for at least 5 years at sites in the original Hughes Ck experiment (Lancaster and Downes 2021). Managers will, however, need to weigh up some potential disadvantages of stakes relative to other management objectives, such as any impacts on public amenity and recreation. In these latter cases, stakes could be trialled over short periods to test whether more substantial investment in improving retentiveness (e.g. addition of large volumes of woody debris) is warranted. Strategic use of large logs in conjunction with multiple pairs of stakes may provide the best chance for improving species diversity across a range of taxa inhabiting degraded rivers.

## Supplementary Information

Below is the link to the electronic supplementary material.

Supplementary file1 (PDF 1416 KB)

## Data Availability

Data will be made available from the University of Melbourne Repository at https://melbourne.figshare.com/.
