# Supplementary material for: A large-scale field experiment across six rivers illustrates how the effects of resource enrichment are context dependent
Source: Oecologia. 2023 May 3;202(1):41–54. doi: 10.1007/s00442-023-05368-z (PMC10229716; doi:10.1007/s00442-023-05368-z)
Supplement: Supplementary file 1 — Supplementary file1 (PDF 1416 KB) [file 442_2023_5368_MOESM1_ESM.pdf]

Electronic Supplementary Material for the article:

A large-scale field experiment across six rivers illustrates how the effects of resource enrichment are context dependent

William D. Bovill[1,2], Barbara J. Downes[1], Nick R. Bond[3], Paul Reich[4], Rhys Coleman[5] and P.S. Lake[6]

[1]*School of Geography, Earth and Atmospheric Sciences, University of Melbourne, Parkville, Victoria, Australia*

[2]*Department of Infrastructure Engineering, University of Melbourne, Parkville, Victoria, Australia*

[3]*Centre for Freshwater Ecosystems, La Trobe University, Wodonga, Victoria, Australia*

[4]*Victorian Department of Environment, Land, Water and Planning, Melbourne, Victoria, Australia*

[5]*Melbourne Water, Docklands, Victoria, Australia*

[6]*School of Biological Sciences, Monash University, Clayton, Victoria, Australia*

Corresponding author: William D. Bovill, Department of Infrastructure Engineering, The University of Melbourne, Parkville, VIC 3010, Australia. wbovill@unimelb.edu.au

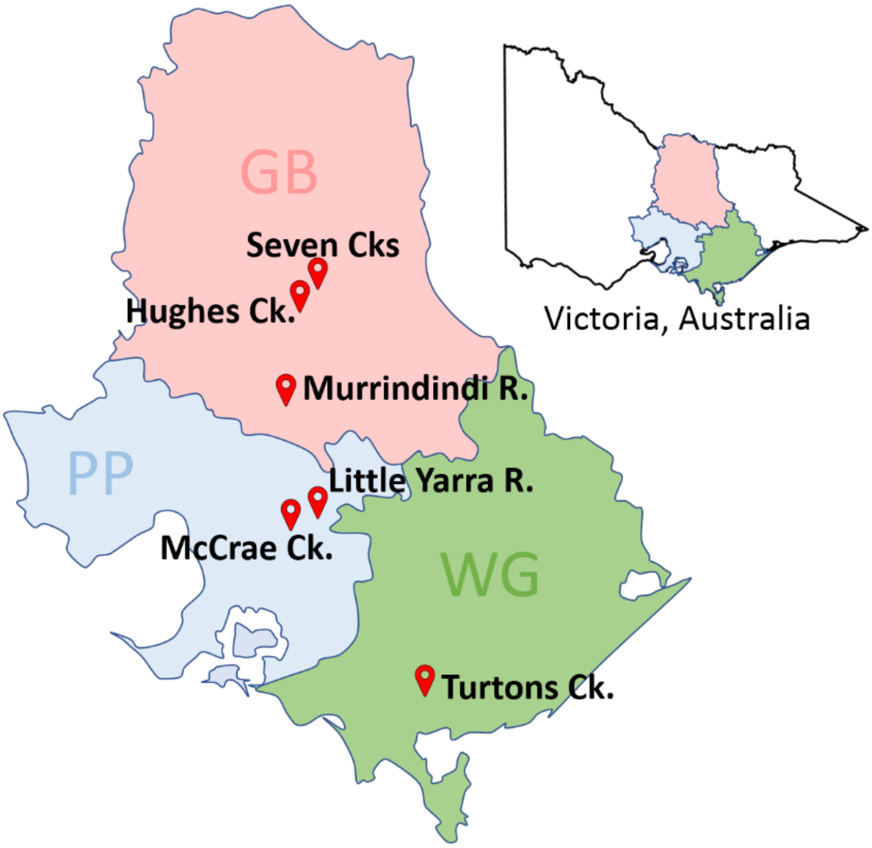

Fig. S1 A map showing rivers that were selected for the experiment, which were based on the following criteria: (1) at least some riparian vegetation must be present along channel sections where experimental sites would be located; (2) reaches upstream of the experimental section must have relatively high cover of riparian vegetation (e.g. average width of vegetation in riparian zone > 50 m) as a source of drifting detritus and a prospective source of invertebrate colonists; (3) the channel should have no obvious human impacts (e.g. heavy stock access, weirs) and flow must be perennial and without poor water quality (e.g. not highly saline); (4) experimental sections should be ~ 6 -10 km long to ensure that all sites would occur within the dispersal range of potential source populations upstream. Criteria 1-3 were assessed with a preliminary desktop exercise using vegetation and hydrological data from a state-wide assessment of river condition (Victorian Department of Environment Land Water & Planning (DELWP) 2010). We followed up with an extensive scoping campaign involving site visits to a shortlist of 37 rivers, but identified just six rivers that fulfilled all selection criteria. Other potential rivers were too dry or did not fulfil the criteria over a sufficient length of channel to accommodate our experimental design

Table S1 For experimental sites (*n* = 6) in each river: the overall reach length, mean width and area of the wetted channel; composition of, and type and number of, stakes installed to achieve a constant density of 0.4 stakes / m^2

River	Reach length (km)	Mean (SD) channel wet width (m)	Mean site area (m^2)	Composition of substrata	Stake type	Stakes / site	
						Mean No.	No. / m^2
Seven Cks.	8.10	7.23 (1.31)	303.5	Sand: 87%<br>Gravel: 5%<br>Cobble: 7%	Wood	122.0	0.4
Hughes Ck.	6.26	9.58 (1.79)	401.3	Sand: 88%<br>Gravel: 6%<br>Cobble: 6%	Wood & Rebar	158.0	0.4
Murrindindi R.	11.49	9.22 (0.67)	390.6	Sand: 43%<br>Gravel: 50%<br>Cobble: 7%	Rebar	156.7	0.4
Little Yarra R.	6.99	4.04 (0.66)	148.3	Sand: 100%<br>Gravel: 0%<br>Cobble: 0%	Wood	60.0	0.4
McCrae Ck.	5.25	3.21 (0.41)	127.5	Sand: 95%<br>Gravel: 4%<br>Cobble: 1%	Wood	51.3	0.4
Turtons Ck.	5.97	4.50 (1.66)	169.5	Clay: 22%<br>Gravel: 65%<br>Cobble: 13%	Rebar	68.7	0.4

Table S2 Dates on which pre-manipulation surveys were conducted and stakes added to manipulation sites, plus the dates of the post-manipulation surveys and sample collection

	**Rivers**					
**Sites**	**Seven Cks.**	**Hughes Ck.**	**Murrindindi R.**	**Little Yarra R.**	**McCrae Ck.**	**Turtons Ck.**
			**Pre manipulation survey**			
Ref. site	14-Dec-2016	21-Dec-2016	15-Dec-2016	08-Dec-2016	07-Dec-2016	02-Jan-2017
Site 1	14-Dec-2016	21-Dec-2016	15-Dec-2016	07-Dec-2016	05-Dec-2016	02-Jan-2017
Site 2	14-Dec-2016	21-Dec-2016	15-Dec-2016	07-Dec-2016	05-Dec-2016	02-Jan-2017
Site 3	14-Dec-2016	22-Dec-2016	15-Dec-2016	06-Dec-2016	05-Dec-2016	03-Jan-2017
Site 4	13-Dec-2016	22-Dec-2016	16-Dec-2016	06-Dec-2016	07-Dec-2016	03-Jan-2017
Site 5	13-Dec-2016	22-Dec-2016	16-Dec-2016	06-Dec-2016	29-Nov-2016	03-Jan-2017
Site 6	13-Dec-2016	22-Dec-2016	16-Dec-2016	06-Dec-2016	08-Dec-2016	03-Jan-2017
			**Post manipulation survey**			
Ref. site	15-Nov-2017	13-Nov-2017	27-Nov-2017	21-Nov-2017	16-Nov-2017	23-Nov-2017
Site 1	14-Nov-2017	14-Nov-2017	28-Nov-2017	21-Nov-2017	20-Nov-2017	23-Nov-2017
Site 2	15-Nov-2017	13-Nov-2017	27-Nov-2017	21-Nov-2017	20-Nov-2017	23-Nov-2017
Site 3	15-Nov-2017	13-Nov-2017	29-Nov-2017	21-Nov-2017	20-Nov-2017	24-Nov-2017
Site 4	14-Nov-2017	13-Nov-2017	28-Nov-2017	22-Nov-2017	22-Nov-2017	24-Nov-2017
Site 5	14-Nov-2017	14-Nov-2017	28-Nov-2017	21-Nov-2017	22-Nov-2017	23-Nov-2017
Site 6	15-Nov-2017	14-Nov-2017	27-Nov-2017	21-Nov-2017	30-Nov-2017	24-Nov-2017

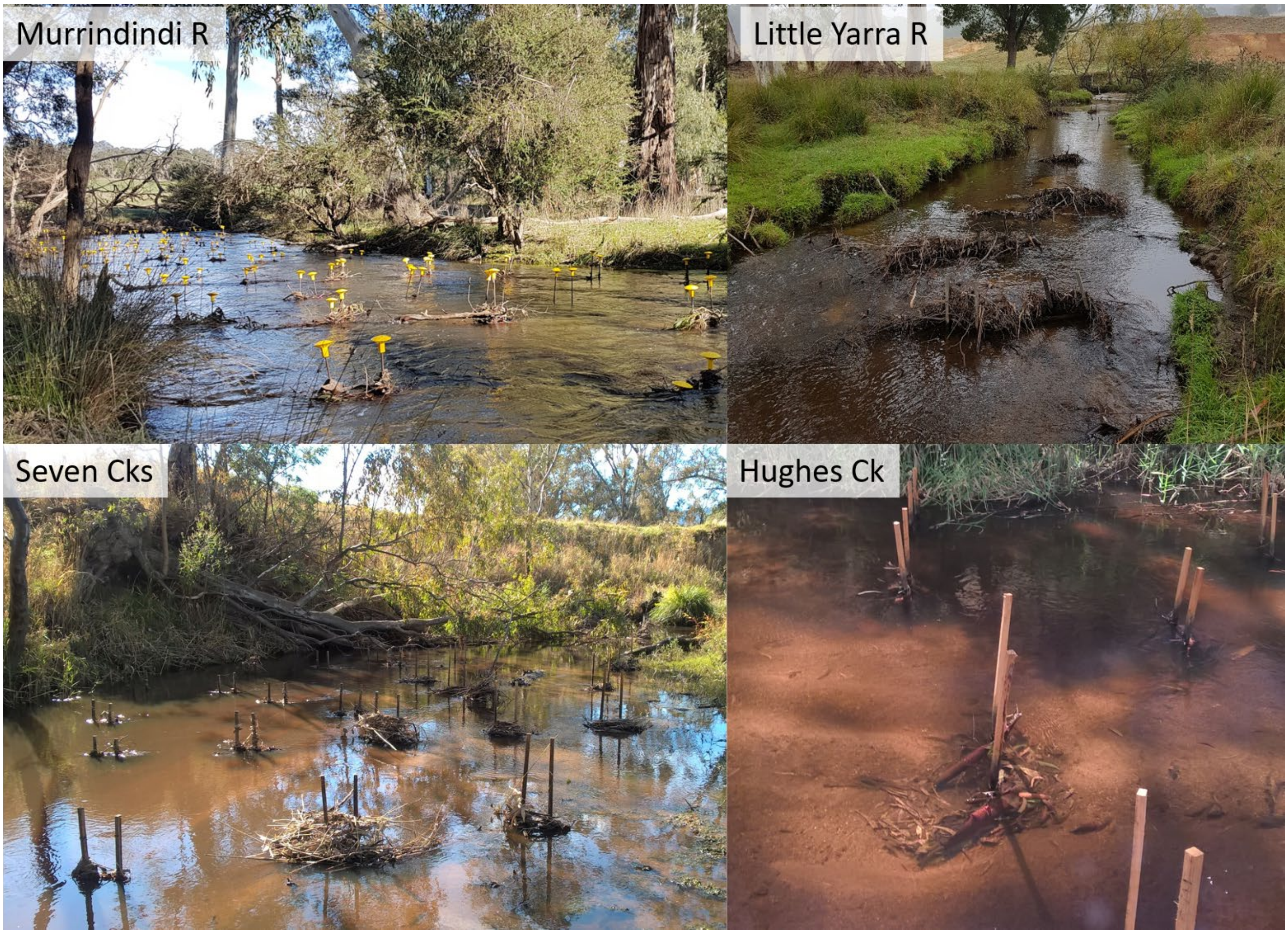

Fig. S2 Photographs of representative sites showing pairs of stakes positioned ~0.3 m apart, perpendicular to flow. Pairs were distributed haphazardly throughout the full length and width of the channel at a density of 0.4 stakes / m^2. Rebar stakes with safety caps (see Murrindindi R.) were deployed at sites with rocky substrata. Wooden stakes were used elsewhere. Photographs are representative of the amounts of detritus accumulated on stakes at all sites after 1 month (Hughes Ck photograph) and at the end of the experiment (all other photographs). Note the large accumulations on stakes in the Murrindindi and Little Yarra rivers, even though they did not show an overall increase in detritus densities at manipulation sites

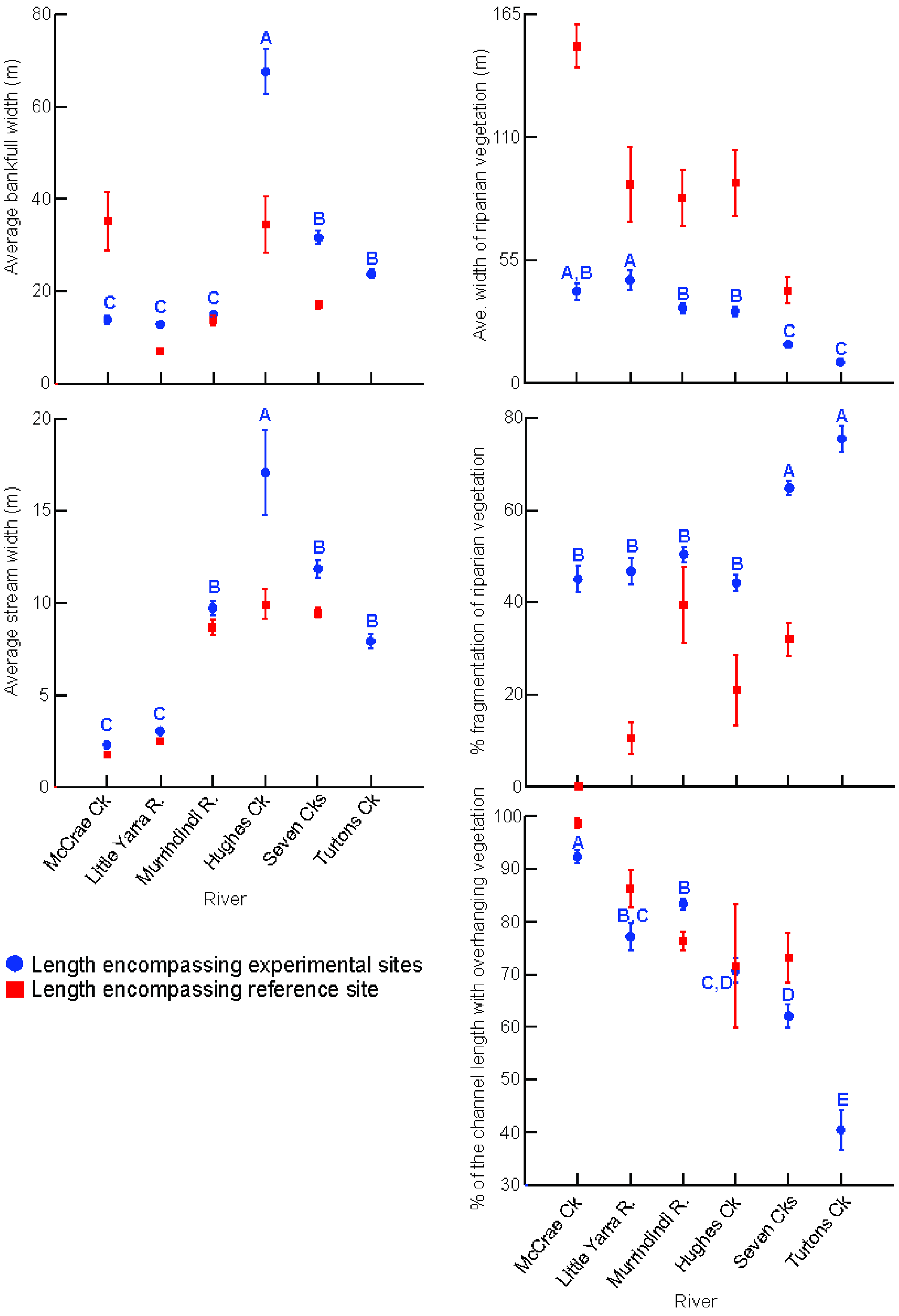

Fig. S3 Channel characteristics (left) and terrestrial vegetation cover (right) of the river length encompassing the experimental sites in six rivers (blue) and 300 m encompassing the upstream, reference site in each of six rivers (red). Both types of variables were measured over 100 m lengths ($n = 3 \times 100$ m lengths for reference reaches; $n = 52 - 115 \times 100$ m lengths for experimental reaches). Channel characteristics are average bankfull width (m) and average width of the stream (m). Vegetation variables are: average width of riparian

vegetation (to a maximum of 200 m); % fragmentation of riparian vegetation (measured as the total proportion of gaps); and the amount of overhanging vegetation (% of channel length in which vegetation hangs over at least part of the wet channel). Data were downloaded from the state-wide assessment of river condition (Victorian Department of Environment Land Water & Planning (DELWP) 2010) but note that these data were unavailable for the reference site at Turtons Ck. Error bars are SE. Rivers that share a letter above the mean value for the experimental length are not significantly different (Tukey's Honestly Significant Difference Test)

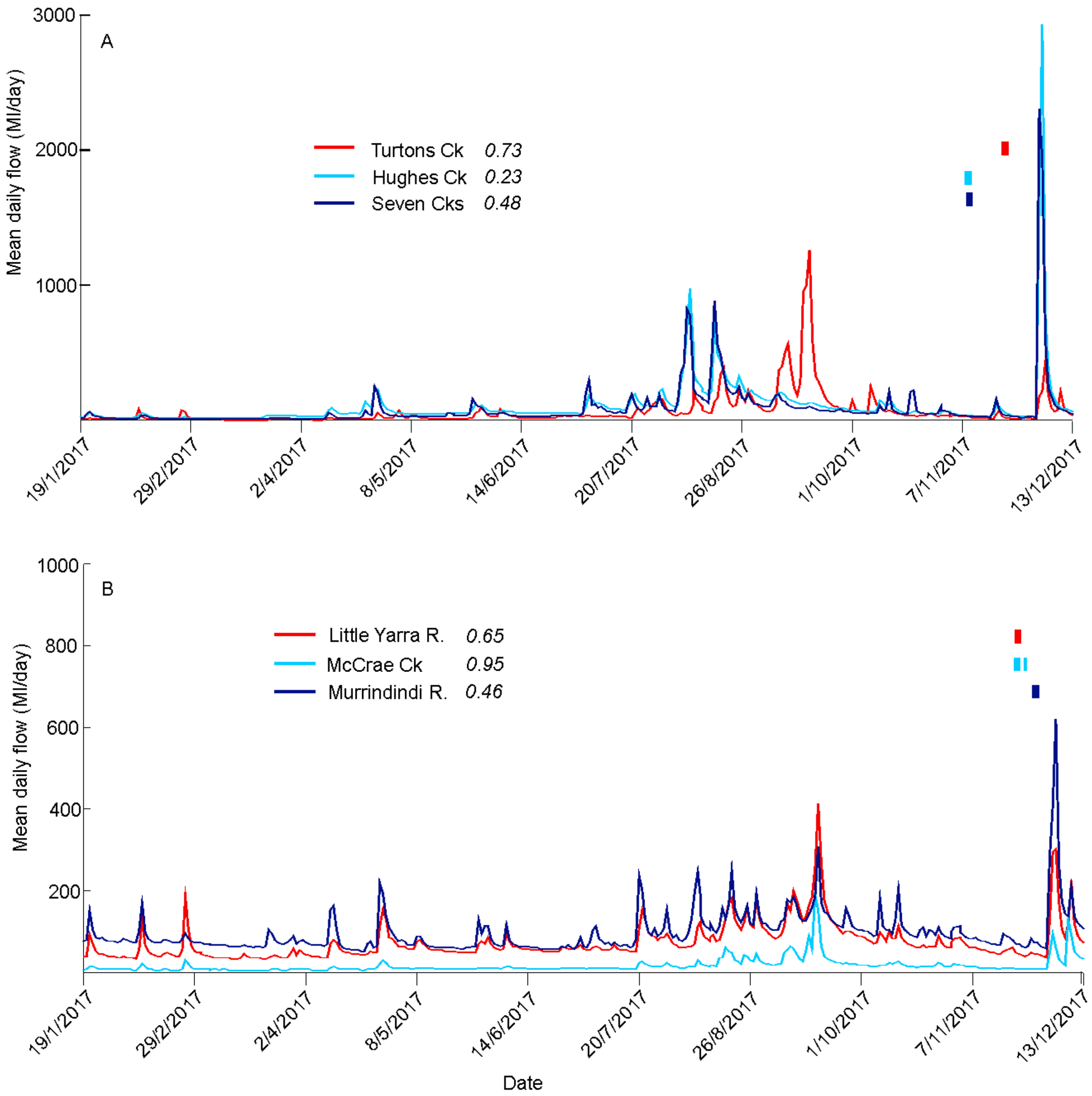

Fig. S4 Average daily discharges during the experimental period in (A) Turtons Ck (using Tarwin River East Branch at Dumbalk North), Hughes Ck (at Tarcombe Road) and Seven Cks (at downstream of Polly McQuinn Weir) creeks and (B) Little Yarra R. (at Yarra Junction) and Murrindindi R. (above Colwells) rivers and McCrae Ck (at Yellingbo). For each relevant gauge, the largest discharge event during 2017 up to the date of final sampling was calculated as a proportion of the size of discharge that has a 2-year average recurrence interval (ARI) for that river, which is approximately bankfull in many streams (Gordon et al. 2004). These proportions are reported next to the name of each river or creek. Data were sourced from Victorian Dept. of Environment, Land, Water & Planning (http://data.water.vic.gov.au/static.htm). The coloured rectangles above the hydrographs indicate the period when the experiment was sampled, with the colour matching that of the river's hydrograph

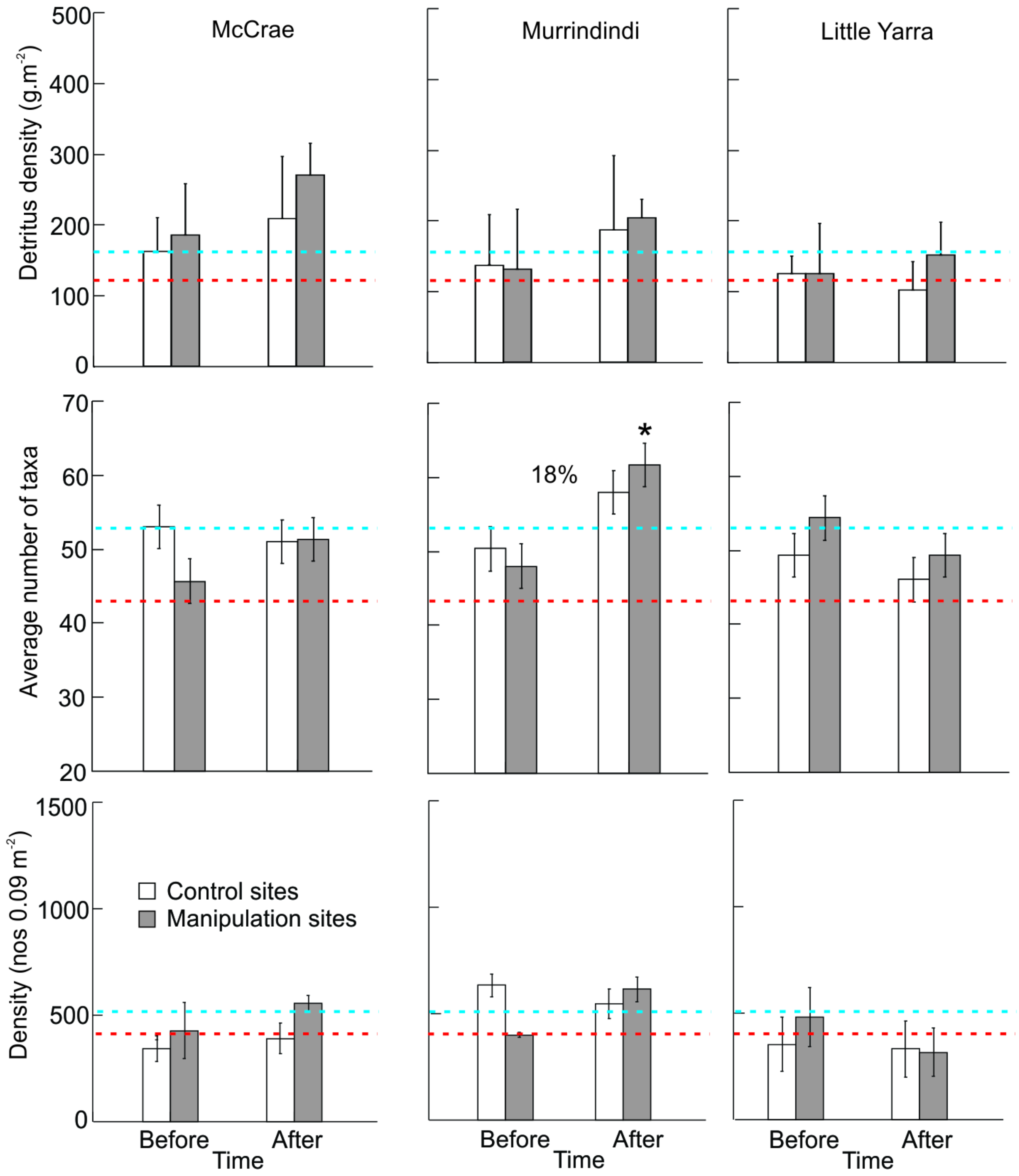

Fig. S5 Average standing stocks of detritus, numbers of taxa and invertebrate densities at control (n = 3) and manipulation (n = 3) sites plotted both before and after manipulation for McCrae Creek, Murrindindi R. and Little Yarra R. Error bars are standard errors calculated from raw data. Also plotted are the 0.8 (red dotted line) and 1.0 (blue dotted line) bio-equivalence values from reference sites. An asterisk indicates that manipulation sites following treatment were significantly different from manipulation and control sites before treatment plus control sites after treatment (see results of contrasts in Table 1). A percentage gives the relevant effect size

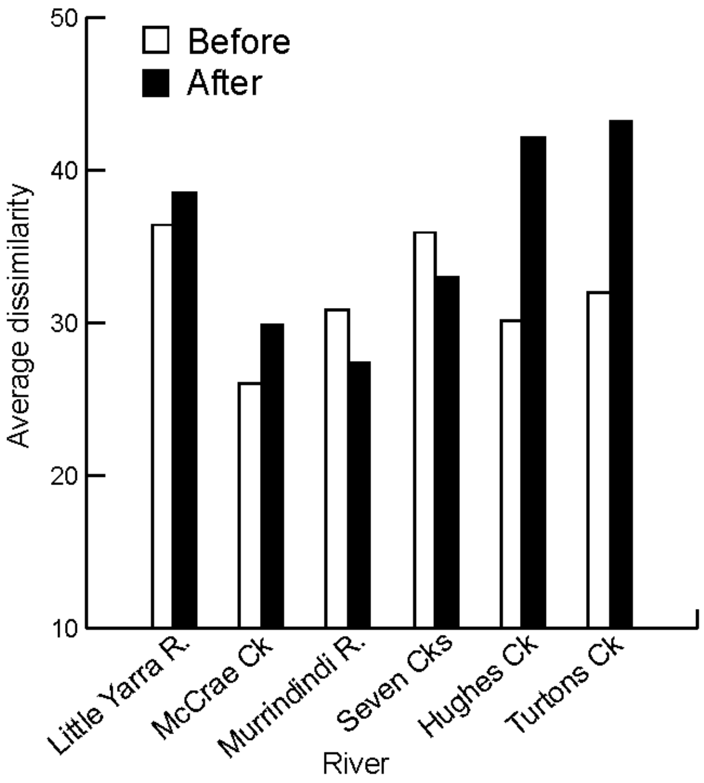

Fig. S6 The average dissimilarity between control (n = 3) and manipulation (n = 3) sites before treatment (open bars) and after treatment (closed bars) for each of the six creeks

Table S3 Comparison of the similarity of control (n = 3) and treatment (n = 3) sites (following treatment) to the reference site (n = 1) for each of three rivers. If manipulation sites increase species richness via drift of individuals from the reference area upstream, then manipulation sites should be more similar to the reference site than are the control sites. Reported for each river are: (1) the mean (and standard deviation, SD) of values of the Bray-Curtis (B-C) similarity coefficient, which was used to measure the similarity of each replicate control or manipulation site to the reference site; (2) the ratio of treatment mean to the control mean; and (3) the 90% confidence interval around the estimate of the ratio, which incorporates variation in each of the mean estimates. The confidence intervals allow a one-tailed test of the hypothesis that the ratio is ≤ 1. This hypothesis is accepted for Seven and Turtons creeks because the CI contains 1, but is rejected for Hughes Creek. Thus, we conclude that the ratio for Hughes Creek is > 1 (the alternative hypothesis). Additionally, we provide the same values from the original Hughes Ck experiment (Lancaster & Downes 2017). The similarities of downstream to upstream sites (which are equivalent to reference sites) are compared for manipulation and control sites, producing a ratio of 1.329. That experiment ran for 365 days, in comparison to the experiment in this study, which ran for 327 days. Correction for the differing length of times produces a ratio of 1.19, which falls within the confidence interval for Hughes Ck, suggesting that manipulation sites at Hughes Ck became as similar to upstream reference sites as did those in the original Hughes Ck experiment. Confidence intervals were calculated following the method of Fieller (Motulsky 1995) and done using the calculator at GraphPad (https://www.graphpad.com/quickcalcs/ErrorProp1.cfm)

**River**	**B-C similarity values, mean ± SD**	**Ratio: manipulation *vs* control**	**90% confidence interval**
Hughes Ck			
Manipulation	67.193 ± 2.407	1.121	1.056 – 1.191
Control	59.930 ± 1.994		
Seven Cks			
Manipulation	62.543 ± 4.982	0.981	0.866 – 1.106
Control	63.745 ± 3.728		
Turtons Ck			
Manipulation	55.804 ± 7.054	0.978	0.821 – 1.138
Control	57.060 ± 2.069		
Hughes Ck in 2014			**Ratio for 327 days**
Manipulation	59.064 ± 5.225	1.329	1.190
Control	44.453 ± 6.308		

Table S4 The taxa contributing (to a total maximum of 60%) to dissimilarity between manipulation and control sites in Hughes Creek following experimental treatment, in order of contributing percentage. Taxa are identified by order and family and then in some cases by genus and species. Manip: average densities (log-transformed) in manipulation sites; Cont: average densities (log-transformed) in control sites; (L) = larvae; (A) = adults; Diss: average dissimilarity; Diss/SD: average dissimilarity divided by standard deviation (larger numbers signal effects that are most consistent across sites); Cont. %: contributing percentage; Cum. %: cumulative percentage. Manipulation or control numbers in bold indicate which of the pair are larger for that taxon

**Order/Family**	**Taxon**	**Manip**	**Cont**	**Diss**	**Diss/SD**	**Cont. %**	**Cum. %**
1. Ephemeroptera/Leptophlebiidae	*Nousia* spp.	**4.76**	1.19	1.51	2.22	3.58	3.58
2. Coleoptera/Elmidae	*Notriolus* early instars	**4.66**	1.63	1.28	1.73	3.05	6.63
3. Trichoptera/Hydroptilidae	*Hydroptila scamandra*	**2.87**	0.78	1.07	1.45	2.54	9.17
4. Odonata/Telephlebiidae	*Austroaeschna* sp. small	**2.38**	0	1	7.52	2.38	11.56
5. Trichoptera/Hydropsychidae	*Asmicridea* sp. AV1	**2.46**	0	1	1.33	2.37	13.93
6. Diptera/Simuliidae	All Simuliidae small	**2.32**	0	0.94	1.33	2.24	16.17
7. Coleoptera/Gyrinidae	Gyrinidae (L)	**2.21**	0	0.92	9.24	2.19	18.36
8. Coleoptera/Elmidae	*Notriolus maculatus* (L)	**2.76**	0.59	0.91	1.9	2.17	20.53
9. Plecoptera/Gripopterygidae	*Illiesoperla australis*	**2.07**	0	0.87	4.68	2.07	26.74
10. Plecoptera/Gripopterygidae	*Leptoperla* sp.	**2.08**	0	0.87	8.52	2.07	24.68
11. Coleoptera/Hydrophilidae	All Hydrophilidae (L)	**2.09**	1.41	0.87	1.31	2.07	22.61
12. Diptera/Chironomidae	*Harrisius* sp.	**4.54**	2.5	0.86	3.58	2.05	28.79
13. Ephemeroptera/Leptophlebiidae	All Leptophlebiidae small	**4.15**	2.15	0.84	2.62	1.99	30.78
14. Diptera/Chironomidae	*Chironomus* sp.	1.3	**2.4**	0.83	1.52	1.98	34.75
15. Trichoptera/Hydroptilidae	All Hydroptilidae small	**2.83**	0.78	0.84	1.53	1.98	32.76
16. Trichoptera/Hydropsychidae	*Cheumatopsyche* small	**4.9**	2.96	0.81	1.51	1.93	36.68
17. Trichoptera/Leptoceridae	All Leptoceridae small	**2.56**	0.78	0.76	1.63	1.8	38.47
18. Ephemeroptera/Baetidae	*Offadens* spp.	3.75	**4**	0.73	1.51	1.74	40.21
19. Diptera/Simuliidae	*Austrosimulium furiosum*	**1.99**	0.7	0.72	1.3	1.72	41.93
20. Trichoptera	Trichoptera small	**1.67**	0	0.72	1.14	1.71	45.34
21. Trichoptera/Hydrobiosidae	*Taschorema evansi*	**2.29**	0.59	0.72	1.62	1.71	43.64
22. Coleoptera/Elmidae	*Notriolus maculatus* (A)	**1.74**	0	0.71	1.3	1.69	47.03
23. Diptera/Simuliidae	*Austrosimulium* small	**1.91**	0.59	0.71	1.38	1.68	48.71
24. Diptera/Chironomidae	Chironominae	**8.8**	7.25	0.67	1.29	1.59	50.29
25. Diptera/Chironomidae	Orthocladinae	**7.71**	6.14	0.64	2.18	1.53	51.82
26. Plecoptera/Gripopterygidae	*Dinotoperla thwaitesi*	**2.08**	0.59	0.63	1.61	1.49	53.31
27. Trichoptera/Hydroptilidae	*Hydroptila* sp.	**1.38**	0	0.62	0.67	1.46	54.77
28. Trichoptera/Hydroptilidae	*Oxyethira* sp.	**1.37**	0	0.6	1.28	1.42	56.19
29. Ephemeroptera/Caenidae	*Tasmanocoenis* sp.	4.5	**4.93**	0.6	1.35	1.41	57.6
30. Coleoptera/Elmidae	*Coxelmis* sp. (L)	**1.37**	0	0.57	1.32	1.36	58.96
31. Trichoptera/Ecnomidae	*Ecnomus* small	**1.67**	1.37	0.57	1.23	1.35	60.32

Table S5 The taxa contributing (to a total maximum of 60%) to dissimilarity between manipulation and control sites in Turtons Creek following experimental treatment, in order of contributing percentage. Taxa are identified by order and family and then in some cases by genus and species. Manip: average densities (transformed) in manipulation sites; Cont: average densities (transformed) in control sites; (L) = larvae; (A) = adults; Diss: average dissimilarity; Diss/SD: average dissimilarity divided by standard deviation (larger numbers equal effects that are most consistent across sites); Cont. %: contributing percentage; Cum. %: cumulative percentage. Manipulation or control numbers in bold indicate which of the pair are larger for that taxon

**Order/Family**	**Taxon**	**Manip**	**Cont**	**Diss**	**Diss /SD**	**Cont. %**	**Cum. %**
1. Trichoptera/Hydropsychidae	*Asmicridea* sp. AV1	**5.04**	3.77	1.15	2.02	2.67	2.67
2. Coleoptera/Elmidae	*Austrolimnius hebrus* (A)	**3.47**	1	0.81	1.58	1.88	4.55
3. Coleoptera/Elmidae	*Notriolus* small	**4.95**	2.45	0.77	2.83	1.78	6.32
4. Diptera/Simuliidae	*Austrosimulium furiosum*	2.34	**2.99**	0.7	1.13	1.61	7.94
5. Coleoptera/Elmidae	*Notriolus quadriplagiatus* (A)	**2.29**	0	0.68	5.09	1.58	9.52
6. Trichoptera/Hydroptilidae	All Hydroptilidae small	0	**2.25**	0.68	5.84	1.58	11.1
7. Trichoptera/Leptoceridae	*Notalina bifaria*	**3.82**	1.67	0.67	1.25	1.56	12.65
8. Coleoptera/Elmidae	*Notriolus setosus* (L)	**3.69**	1.41	0.67	2.31	1.54	14.2
9. Trichoptera/ Conoesucidae	All Conoesucidae	**2.65**	2.16	0.65	1.19	1.5	15.7
10. Diptera/Simuliidae	All Simuliidae small	2.7	**2.95**	0.65	1.72	1.49	17.19
11. Coleoptera/Elmidae	*Notriolus quadriplagiatus* (L)	**2.88**	0.84	0.64	1.53	1.48	18.67
12. Trichoptera/Leptoceridae	*Triplectides similis*	**2**	0	0.61	4.69	1.42	20.08
13. Diptera/Simuliidae	*Austrosimulium* small	2.04	**2.32**	0.6	1.31	1.38	21.47
14. Trichoptera/Hydrobiosidae	All Hydrobiosidae small	**2.5**	0.59	0.59	1.87	1.37	22.84
15. Coleoptera/Elmidae	Notriolus victoriae (L)	**2.51**	0.59	0.58	1.8	1.34	24.18
16. Ephemeroptera/Leptophlebiidae	*Nousia* spp.	**5.43**	3.76	0.56	1.17	1.3	25.48
17. Hemiptera/Corixidae	*Micronecta* spp.	2.42	**3.59**	0.56	1.05	1.29	26.77
18. Trichoptera/Ecnomidae	*Ecnomus russellius*	**2.28**	1.48	0.55	1.29	1.28	28.05
19. Trichoptera/Leptoceridae	*Oecetis* spp.	**2.16**	0.84	0.55	1.15	1.27	29.31
20. Trichoptera/Hydrobiosidae	*Taschorema evansi*	**2.13**	0.7	0.55	1.21	1.26	30.57
21. Trichoptera/Ecnomidae	*Ecnomus continentalis*	0.96	**2.26**	0.54	1.5	1.25	33.08
22. Mollusca	Bivalvia	**2.97**	2.38	0.54	1.34	1.25	31.83
23. Coleoptera/Elmidae	*Simsonia* sp. L2E(A) / *angusta* larva	1.37	**2.21**	0.53	1.38	1.23	34.31
24. Ephemeroptera/Leptophlebiidae	*Thraulophlebia* sp.	**3.13**	2.75	0.53	1.55	1.23	35.54
25. Coleoptera/Elmidae	*Austrolimnius resa* (L)	1.1	**1.7**	0.52	1.18	1.21	36.75
26. Plecoptera/Gripopterygidae	*Dinotoperla brevipennis*	**1.52**	0	0.47	1.3	1.08	37.83
27. Diptera/Empididae	All Empididae	**2.66**	1.3	0.46	1.15	1.07	38.89
28. Trichoptera/Calamoceratidae	*Anisocentropus* sp.	**1.91**	1.67	0.46	1.19	1.06	39.96
29. Coleoptera/Ptilodactylidae	*Byrrocryptus* sp.	**1.37**	0	0.44	1.2	1.02	40.98
30. Diptera/Chironomidae	*Chironomus* sp.	**1.15**	1.05	0.43	0.86	1	41.98
31. Trichoptera/Philorheithridae	All Philorheithridae small	0.59	**1.67**	0.43	1.27	1	42.97
32. Coleoptera/Elmidae	*Austrolimnius resa* (A)	**1.67**	0.59	0.43	1.14	0.99	43.96
33. Trichoptera/Hydrobiosidae	*Apsilochorema gisbum*	**1.52**	0	0.42	1.24	0.98	44.94
34. Trichoptera/Hydrobiosidae	*Taschorema* spp. small	**1.92**	1.72	0.42	0.98	0.97	45.91
35. Mollusca/Tateidae	Tateidae	**1.3**	0	0.41	1.31	0.94	46.85

36. Diptera/Chironomidae	*Harrisius* sp.	**2.02**	0.59	0.4	1.53	0.94	47.78
37. Coleoptera/Elmidae	*Austrolimnius hebrus* (A)	**2.24**	1.84	0.4	1.34	0.93	48.72
38. Coleoptera/Elmidae	*Austrolimnius* spp. small	0.84	**1.3**	0.39	1.28	0.91	49.63
39. Ephemeroptera/Leptophlebiidae	*Ulmerophlebia* sp. AV2	**1.65**	1.43	0.39	1.27	0.91	50.54
40. Ephemeroptera/Leptophlebiidae	*Atalophlebia* small	**1.56**	1.48	0.39	1.06	0.91	51.45
41. Coleoptera/Psephenidae	*Sclerocyphon striatus*	**2.62**	1.63	0.39	0.96	0.9	52.36
42. Trichoptera/Hydrobiosidae	*Apsilochorema* sp. small	**1.54**	0.59	0.39	1.25	0.9	53.26
43. Plecoptera/Gripopterygidae	*Illiesoperla australis*	**2.41**	1.19	0.39	1.18	0.9	54.16
44. Trichoptera/Leptoceridae	*Notalina* spp. small	**0.93**	0.84	0.39	0.84	0.89	55.05
45. Megaloptera/Corydalidae	*Archichauliodes* sp	**1.71**	1.41	0.38	1.09	0.89	55.94
46. Ephemeroptera/Leptophlebiidae	*Austrophlebioides* sp.	4.01	**4.7**	0.38	1.3	0.88	56.82
47. Trichoptera/Atriplectidae	*Atriplectides* sp.	**1.49**	0.78	0.38	1.11	0.88	57.7
48. Coleoptera/Elmidae	*Coxelmis novemnotata* (L)	**1.37**	0	0.38	1.29	0.88	58.59
49. Plecoptera/Gripopterygidae	*Dinotoperla thwaitesi*	**1.43**	0.59	0.38	1.15	0.87	60.33

Table S6 Outcomes of tests for treatment effects on the most common taxa (taxa sufficiently abundant to permit the analysis) in Hughes Ck using analysis of variance on the full data set followed by the *a priori* contrast (see Statistical Analysis section) within the Time x Treatment x River term to test for responses in Hughes Ck. Reported below is the taxonomic classification and species name (where relevant), the effect size (calculated as explained in Statistical Analyses in the Methods), F-value and P-value to test for a treatment effect. All taxa in bold and had statistically significant outcomes. Effect sizes are shown in blue where a density increase was recorded at manipulation sites, and in red where a relative decrease was recorded. NEW: taxon had zero abundance in control sites and manipulation sites before treatment; LOST: taxon had zero abundance at manipulation sites after treatment; (L) = larvae; (A) = adults. The column labelled Com. indicates for those taxa (31 taxa) whether the taxon was a responder (R) or non-responder (NR) in the previous experiment in Hughes Ck (Lancaster and Downes 2017). Two-thirds of both responders and non-responders were consistent with the previous experiment

Order /Class	Family	Taxon	Effect size (%)	F	P	Com.
1. Mollusca	Planorbidae	*Ferrissia petterdi*	121	8.848	**0.007**	R
2. Mollusca	Planorbidae	*Ferrissia tasmanica*	129	9.344	**0.005**	
3. Ephemeroptera	Baetidae	*Offadens* spp.	-11	1.216	0.281	R
4. Ephemeroptera	Caenidae	*Tasmanocoenis* sp.	-7	0.802	0.379	NR
5. Ephemeroptera	Leptophlebiidae	*Atalophlebia* sp. AV2	109	0.693	0.413	
6. Ephemeroptera	Leptophlebiidae	*Atalophlebia* sp. AV9	LOST	8.213	**0.009**	
7. Ephemeroptera	Leptophlebiidae	*Atalophlebia* small	136	0.589	0.450	
8. Ephemeroptera	Leptophlebiidae	*Nousia* spp.	360	48.495	**<0.001**	R
9. Ephemeroptera	Leptophlebiidae	*Ulmerophlebia* small	32	0.038	0.847	NR
10. Ephemeroptera	Leptophlebiidae	All Leptophlebiidae small	38	27.783	**<0.001**	
11. Plecoptera	Gripopterygidae	*Dinotoperla thwaitesi*	1011	24.570	**<0.001**	R
12. Plecoptera	Gripopterygidae	*Illiesoperla australis*	NEW	16.579	**<0.001**	R
13. Plecoptera	Gripopterygidae	*Leptoperla* sp.	NEW	16.909	**<0.001**	R
14. Odonata	Gomphidae	*Austrogomphus cornutus*	281	6.042	**0.022**	
15. Odonata	Telephlebiidae	*Austroaeschna* sp. small	NEW	39.922	**<0.001**	
16. Hemiptera	Corixidae	*Micronecta* spp.	4	0.333	0.569	NR
17. Diptera	Ceratopogonidae	*Forcipomyia* sp.	330	3.963	0.058	
18. Diptera	Ceratopogonidae	All Ceratopogonidae	82	5.679	**0.025**	NR
19. Diptera	Chironomidae	*Chironomus* sp.	-7	0.008	0.931	NR
20. Diptera	Chironomidae	*Harrisius* sp.	192	26.664	**<0.001**	R
21. Diptera	Chironomidae	*Stempellina* sp.	-37	0.284	0.599	
22. Diptera	Chironomidae	Chironominae	21	11.535	**0.002**	NR
23. Diptera	Chironomidae	Orthocladinae	22	34.196	**<0.001**	NR
24. Diptera	Chironomidae	Tanypodinae	-2	0.062	0.806	
25. Diptera	Chironomidae	Other Chironomidae	LOST	3.466	0.075	
26. Diptera	Empididae	All Empididae	12	1.721	0.202	NR

27.	Diptera	Psychodidae	All Psychodidae	NEW	9.427	**0.005**	
28.	Diptera	Simuliidae	*Austrosimulium furiosum*	760	11.209	**0.003**	R
29.	Diptera	Simuliidae	*Austrosimulium* small	428	6.218	**0.020**	
30.	Diptera	Simuliidae	All Simuliidae small	NEW	18.350	**<0.001**	
31.	Diptera	Tipulidae	All Tipulidae	125	3.711	0.066	NR
32.	Coleoptera	Elmidae	*Austrolimnius waterhousei* (A)	2	0.002	0.962	NR
33.	Coleoptera	Elmidae	*Austrolimnius waterhousei* (L)	3	0.095	0.760	NR
34.	Coleoptera	Elmidae	*Coxelmis novemnotata* (L)	23	0.170	0.684	
35.	Coleoptera	Elmidae	*Coxelmis* sp. (L)	265	20.366	**<0.001**	
36.	Coleoptera	Elmidae	*Notriolus maculatus* (A)	NEW	35.141	**<0.001**	R
37.	Coleoptera	Elmidae	*Notriolus maculatus* (L)	648	40.104	**<0.001**	R
38.	Coleoptera	Elmidae	*Notriolus* small (L)	292	36.278	**<0.001**	R
39.	Coleoptera	Gyrinidae	All Gyrinidae (L)	NEW	54.324	**<0.001**	
40.	Coleoptera	Hydraenidae	*Limnebius* sp. (A)	69	0.718	0.405	
41.	Coleoptera	Hydrophilidae	*Berosus* sp. (L)	281	217.012	**<0.001**	NR
42.	Coleoptera	Hydrophilidae	Hydrophilidae (L)	9	0.095	0.760	
43.	Coleoptera	Psephenidae	*Sclerocyphon* sp.	LOST	1.406	0.247	
44.	Trichoptera	Conoesucidae	All Conoesucidae	-52	2.176	0.153	R
45.	Trichoptera	Ecnomidae	*Ecnomus continentalis*	-50	16.251	**<0.001**	R
46.	Trichoptera	Ecnomidae	*Ecnomus pansus*	-50	3.677	0.067	
47.	Trichoptera	Ecnomidae	*Ecnomus* sp. small	-34	4.382	**0.047**	
48.	Trichoptera	Hydrobiosidae	*Taschorema* complex small	630	5.202	**0.032**	
49.	Trichoptera	Hydrobiosidae	*Taschorema evansi*	515	8.758	**0.007**	
50.	Trichoptera	Hydrobiosidae	*Ulmerochorema lentum*	281	3.742	0.065	
51.	Trichoptera	Hydrobiosidae	*Ulmerochorema rubiconum*	LOST	1.718	0.202	
52.	Trichoptera	Hydrobiosidae	All Hydrobiosidae small	16	0.026	0.874	
53.	Trichoptera	Hydropsychidae	*Asmicridea* sp. AV1	NEW	17.569	**<0.001**	R
54.	Trichoptera	Hydropsychidae	*Cheumatopsyche* sp. AV1	71	1.876	0.183	R
55.	Trichoptera	Hydropsychidae	*Cheumatopsyche* sp. AV2	19	0.778	0.387	R
56.	Trichoptera	Hydropsychidae	*Cheumatopsyche deani*	0	0.000	0.996	R
57.	Trichoptera	Hydropsychidae	All *Cheumatopsyche* small	68	16.860	**<0.001**	
58.	Trichoptera	Hydroptilidae	*Hydroptila scamandra*	500	30.963	**<0.001**	
59.	Trichoptera	Hydroptilidae	*Hydroptila* sp.	NEW	18.000	**<0.001**	
60.	Trichoptera	Hydroptilidae	*Oxyethira* sp.	NEW	25.165	**<0.001**	
61.	Trichoptera	Hydroptilidae	All Hydroptilidae small	147	14.245	**0.001**	
62.	Trichoptera	Leptoceridae	*Oecetis* spp.	-60	2.187	0.152	R
63.	Trichoptera	Leptoceridae	*Triplectides ciuskus*	LOST	4.266	0.050	R
64.	Trichoptera	Leptoceridae	All Leptoceridae small	61	1.864	0.185	
65.	Trichoptera	Trichoptera	Trichoptera very small	NEW	5.916	**0.023**	

Table S7 Outcomes of tests for treatment effects on the most common taxa (taxa with at least 200 individuals) in Turtons Ck using analysis of variance on the full data set followed by an *a priori* contrast within the Time x Treatment x River term ([control and manipulation sites before treatment + control sites after treatment in Turtons Ck] = 3 x [manipulation sites after treatment in Turtons Ck]). Reported is the taxonomic classification and species name (where relevant) and the effect size, where positive values indicate a relative increase at manipulation sites and negative values indicate a relative increase in controls. All taxa in bold had statistically significant outcomes, and those with effect sizes in blue show a relative increase at manipulation sites whereas those in red show a relative increase at controls. NEW: taxon had zero abundance in control sites and manipulation sites before treatment; LOST: taxon had zero abundance at manipulation sites after treatment; (L) = larvae; (A) = adults

Order /Class		Family	Taxon	Effect size (%)	F	P
1.	Mollusca	Bivalvia	All Bivalvia	-12	0.376	0.545
2.	Mollusca	Physidae	*Physella* sp.	LOST	2.410	0.134
3.	Mollusca	Planorbidae	*Ferrissia petterdi*	-16	0.070	0.793
4.	Mollusca	Planorbidae	*Ferrissia tasmanica*	-8	0.012	0.913
5.	Mollusca	Tateidae	All Tateidae	51	1.083	0.308
6.	Amphipoda	Amphipoda	All Amphipoda	-8	0.011	0.916
7.	Ephemeroptera	Baetidae	*Offadens* spp.	-7	0.809	0.377
8.	Ephemeroptera	Caenidae	*Tasmanocoenis* sp.	6	0.068	0.796
9.	Ephemeroptera	Leptophlebiidae	*Nousia* spp.	17	2.062	0.164
10.	Ephemeroptera	Leptophlebiidae	*Austrophlebioides* sp.	2	0.063	0.805
11.	Ephemeroptera	Leptophlebiidae	*Thraulophlebia* sp.	-17	1.054	0.315
12.	Ephemeroptera	Leptophlebiidae	*Atalophlebia* sp. AV5	32	0.515	0.480
13.	Ephemeroptera	Leptophlebiidae	*Atalophlebia* small	9	0.100	0.755
14.	Ephemeroptera	Leptophlebiidae	*Ulmerophlebia* sp. AV1	200	1.421	0.245
15.	Ephemeroptera	Leptophlebiidae	*Ulmerophlebia* sp. AV2	-43	13.560	**0.001**
16.	Ephemeroptera	Leptophlebiidae	*Ulmerophlebia* small	-8	0.209	0.652
17.	Ephemeroptera	Leptophlebiidae	Leptophlebiidae small	1	0.026	0.873
18.	Plecoptera	Gripopterygidae	*Illiesoperla australis*	562	17.594	**<0.001**
19.	Plecoptera	Gripopterygidae	*Dinotoperla christinae*	16	0.084	0.774
20.	Plecoptera	Gripopterygidae	*Dinotoperla thwaitesi*	665	10.628	**0.003**
21.	Plecoptera	Gripopterygidae	*Dinotoperla brevipennis*	NEW	14.920	**0.001**
22.	Plecoptera	Notonemouridae	*Austrocercella mariannae*	20	0.729	0.402
23.	Plecoptera	Gripopterygidae	Gripopterygidae small	373	3.332	0.080
24.	Hemiptera	Corixidae	*Micronecta* spp.	-41	24.742	**<0.001**
25.	Megaloptera	Corydalidae	*Archichauliodes* sp.	167	8.201	**0.009**
26.	Diptera	Ceratopogonidae	All Ceratopogonidae	-29	1.463	0.238
27.	Diptera	Chironomidae	*Chironomus* sp.	-23	0.105	0.748
28.	Diptera	Chironomidae	*Harrisius* sp.	965	11.209	**0.003**

29.	Diptera	Chironomidae	*Stempellina* sp.	-41	1.534	0.228
30.	Diptera	Chironomidae	Chironominae	2	0.138	0.713
31.	Diptera	Chironomidae	Tanypodinae	-14	4.808	**0.038**
32.	Diptera	Chironomidae	Orthocladinae	20	19.047	**<0.001**
33.	Diptera	Empididae	All Empididae	66	12.292	**0.002**
34.	Diptera	Simuliidae	*Austrosimulium furiosum*	15	0.309	0.584
35.	Diptera	Simuliidae	*Austrosimulium* small	-1	0.001	0.972
36.	Diptera	Simuliidae	Simuliidae small	7	0.115	0.738
37.	Diptera	Tipulidae	All Tipulidae	-11	0.125	0.727
38.	Coleoptera	Dytiscidae	Dytiscidae (L)	-69	3.663	0.068
39.	Coleoptera	Dytiscidae	Dytiscidae (A)	LOST	2.162	0.154
40.	Coleoptera	Elmidae	*Austrolimnius hebrus* (L)	-5	0.065	0.801
41.	Coleoptera	Elmidae	*Austrolimnius hebrus* (A)	82	15.780	**0.001**
42.	Coleoptera	Elmidae	*Austrolimnius resa* (L)	-48	14.083	**0.001**
43.	Coleoptera	Elmidae	*Austrolimnius resa* (A)	266	27.098	**<0.001**
44.	Coleoptera	Elmidae	*Austrolimnius dayi* (A)	8	0.007	0.933
45.	Coleoptera	Elmidae	*Austrolimnius fallax* (A)	LOST	4.000	0.057
46.	Coleoptera	Elmidae	*Austrolimnius* spp small (L)	-63	4.598	**0.042**
47.	Coleoptera	Elmidae	*Austrolimnius* (*Limnelmis* subg) (L)	50	0.500	0.486
48.	Coleoptera	Elmidae	*Austrolimnius* spp other (A)	500	8.909	**0.006**
49.	Coleoptera	Elmidae	*Notriolus setosus* (L)	263	96.908	**<0.001**
50.	Coleoptera	Elmidae	*Notriolus quadriplagiatus* (L)	250	20.206	**<0.001**
51.	Coleoptera	Elmidae	*Notriolus quadriplagiatus* (A)	442	10.663	**0.003**
52.	Coleoptera	Elmidae	*Notriolus victoriae* (L)	239	9.608	**0.005**
53.	Coleoptera	Elmidae	*Notriolus maculatus* (L)	365	3.175	0.087
54.	Coleoptera	Elmidae	*Notriolus* small (L)	54	8.969	**0.006**
55.	Coleoptera	Elmidae	*Kingolus flavosignatus* (L)	-62	2.776	0.109
56.	Coleoptera	Elmidae	*Kingolus flavosignatus* (A)	32	0.455	0.506
57.	Coleoptera	Elmidae	*Kingolus aeratus/metallicus* (L)	LOST	3.650	0.068
58.	Coleoptera	Elmidae	*Simsonia* spL2E(A)/ *angusta* (L)	82	2.413	0.133
59.	Coleoptera	Elmidae	*Coxelmis novemnotata* (L)	NEW	27.978	**<0.001**
60.	Coleoptera	Hydraenidae	All Hydraenidae (L)	238	2.827	0.106
61.	Coleoptera	Psephenidae	*Sclerocyphon striatus*	80	9.238	**0.006**
62.	Coleoptera	Psephenidae	*Sclerocyphon* sp.	LOST	30.056	**<0.001**
63.	Coleoptera	Ptilodactylidae	*Byrrocryptus* sp.	NEW	11.985	**0.002**
64.	Coleoptera	Scirtidae	All Scirtidae (L)	64	3.879	0.061
65.	Trichoptera	Atriplectidae	*Atriplectides* sp.	21	0.238	0.630
66.	Trichoptera	Calamoceratidae	*Anisocentropus* sp.	39	1.323	0.261
67.	Trichoptera	Calocidae	All Calocidae small	LOST	8.336	**0.008**
68.	Trichoptera	Conoesucidae	All Conoesucidae	16	0.774	0.388
69.	Trichoptera	Ecnomidae	*Ecnomus russellius*	16	1.127	0.299
70.	Trichoptera	Ecnomidae	*Ecnomus continentalis*	-51	8.171	**0.009**
71.	Trichoptera	Ecnomidae	*Ecnomus tillyardi*	LOST	3.778	0.064
72.	Trichoptera	Ecnomidae	*Ecnomus* sp. small	-73	14.625	**0.001**
73.	Trichoptera	Glossosomatidae	*Agapetus* sp.	LOST	6.936	**0.015**
74.	Trichoptera	Helicopsychidae	*Helicopsyche* sp.	-36	35.039	**<0.001**
75.	Trichoptera	Hydrobiosidae	*Taschorema evansi*	39	0.840	0.369
76.	Trichoptera	Hydrobiosidae	*Taschorema* complex small	5	0.039	0.845
77.	Trichoptera	Hydrobiosidae	*Apsilochorema gisbum*	46	1.933	0.177

78.	Trichoptera	Hydrobiosidae	*Apsilochorema* sp. small	3	0.006	0.939
79.	Trichoptera	Hydrobiosidae	*Ulmerochorema lentum*	-8	0.009	0.927
80.	Trichoptera	Hydrobiosidae	*Ulmerochorema rubiconum*	LOST	2.929	0.100
81.	Trichoptera	Hydrobiosidae	Hydrobiosidae small	253	8.676	**0.007**
82.	Trichoptera	Hydropsychidae	*Asmicridea* sp. AV1	39	3.331	0.080
83.	Trichoptera	Hydropsychidae	*Cheumatopsyche* small	NEW	8.848	**0.007**
84.	Trichoptera	Hydropsychidae	Hydropsychidae small	LOST	2.518	0.126
85.	Trichoptera	Hydroptilidae	*Hellyethira simplex*	LOST	18.036	**<0.001**
86.	Trichoptera	Hydroptilidae	*Hydroptila scamandra*	LOST	2.522	0.125
87.	Trichoptera	Hydroptilidae	Hydroptilidae small	LOST	39.276	**<0.001**
88.	Trichoptera	Leptoceridae	*Notalina bifaria*	251	33.464	**<0.001**
89.	Trichoptera	Leptoceridae	*Oecetis* spp.	95	3.395	0.078
90.	Trichoptera	Leptoceridae	*Notalina spira*	-25	1.337	0.259
91.	Trichoptera	Leptoceridae	*Notalina ordina*	41	0.447	0.510
92.	Trichoptera	Leptoceridae	*Notalina fulva*	LOST	2.568	0.122
93.	Trichoptera	Leptoceridae	*Notalina* spp. small	45	0.223	0.641
94.	Trichoptera	Leptoceridae	*Triplectides ciuskus*	105	1.825	0.189
95.	Trichoptera	Leptoceridae	*Triplectides similis*	962	41.427	**<0.001**
96.	Trichoptera	Leptoceridae	Leptoceridae small	-14	0.325	0.574
97.	Trichoptera	Odontoceridae	*Marilia bola*	330	7.123	**0.013**
98.	Trichoptera	Philorheithridae	*Kosrheithrus tillyardi*	LOST	2.693	0.114
99.	Trichoptera	Philorheithridae	All Philorheithridae small	-66	3.934	0.059
100.	Trichoptera	Trichoptera	Trichoptera very small	-50	0.679	0.418

Table S8 Outcomes of tests for treatment effects on the most common taxa (taxa with at least 200 individuals) in Seven Cks using analysis of variance on the full data set followed by an *a priori* contrast within the Time x Treatment x River term ([control and manipulation sites before treatment + control sites after treatment in Turtons Ck] = 3 x [manipulation sites after treatment in Turtons Ck]). Reported is the taxonomic classification and species name (where relevant) and the effect size, where positive values indicate a relative increase at manipulation sites and negative values indicate a relative increase in controls. All taxa in bold had statistically significant outcomes, and those with effect sizes in blue show a relative increase at manipulation sites whereas those in red show a relative increase at controls. NEW: taxon had zero abundance in control sites and manipulation sites before treatment; LOST: taxon had zero abundance at manipulation sites after treatment; (L) = larvae; (A) = adults

Order /Class	Family	Taxon	Effect size (%)	F	P
1. Mollusca	Bivalvia	Bivalvia	-14	0.047	0.830
2. Mollusca	Physidae	*Physella* sp.	94	0.911	0.349
3. Mollusca	Planorbidae	*Ferrissia petterdi*	-1	0.001	0.973
4. Mollusca	Planorbidae	*Ferrissia tasmanica*	25	0.636	0.433
5. Ephemeroptera	Baetidae	*Offadens* spp.	5	0.331	0.570
6. Ephemeroptera	Caenidae	*Tasmanocoenis* sp.	3	0.145	0.706
7. Ephemeroptera	Leptophlebiidae	*Atalophlebia* small	-21	0.787	0.384
8. Ephemeroptera	Leptophlebiidae	*Atalophlebia* sp. AV9	500	**7.791**	**0.010**
9. Ephemeroptera	Leptophlebiidae	Leptophlebiidae small	21	**15.018**	**0.001**
10. Ephemeroptera	Leptophlebiidae	*Nousia* spp.	84	**22.215**	**<0.001**
11. Ephemeroptera	Leptophlebiidae	*Thraulophlebia* sp.	200	1.504	0.232
12. Ephemeroptera	Leptophlebiidae	*Ulmerophlebia* small	124	3.908	0.060
13. Plecoptera	Gripopterygidae	*Dinotoperla thwaitesi*	1122	**30.265**	**<0.001**
14. Plecoptera	Gripopterygidae	*Illiesoperla australis*	324	**21.289**	**<0.001**
15. Plecoptera	Gripopterygidae	*Riekoperla tuberculata*	NEW	**25.387**	**<0.001**
16. Odonata	Gomphidae	*Austrogomphus guerini*	50	0.500	0.486
17. Odonata	Telephlebiidae	*Austroaeschna* sp. small	736	**20.326**	**<0.001**
18. Odonata	Zygoptera	Zygoptera small	500	**16.667**	**<0.001**
19. Hemiptera	Corixidae	*Micronecta* spp.	0	0.001	0.978
20. Diptera	Ceratopogonidae	Ceratopogonidae	28	0.943	0.341
21. Diptera	Chironomidae	Chironominae	33	**22.358**	**<0.001**
22. Diptera	Chironomidae	Orthocladinae	33	**62.494**	**<0.001**
23. Diptera	Chironomidae	Tanypodinae	8	0.991	0.329
24. Diptera	Chironomidae	*Chironomus* sp.	-4	0.009	0.926
25. Diptera	Chironomidae	*Harrisius* sp.	259	**24.739**	**<0.001**
26. Diptera	Chironomidae	*Stempellina* sp.	182	1.624	0.215
27. Diptera	Empididae	Empididae	-10	1.780	0.195
28. Diptera	Psychodidae	Psychodidae	32	0.108	0.745
29. Diptera	Simuliidae	*Austrosimulium furiosum*	99	**5.724**	**0.025**
30. Diptera	Simuliidae	*Austrosimulium* small	1	<0.001	0.988

31. Diptera	Simuliidae	Simuliidae small	-32	1.429	0.244
32. Diptera	Stratiomyidae	Stratiomyidae	200	**4.937**	**0.036**
33. Diptera	Tipulidae	Tipulidae	-21	0.317	0.579
34. Coleoptera	Dytiscidae	Dytiscidae adult	LOST	0.904	0.351
35. Coleoptera	Dytiscidae	Dytiscidae larva	11	0.027	0.871
36. Coleoptera	Elmidae	*Austrolimnius waterhousei* (A)	17	0.046	0.832
37. Coleoptera	Elmidae	*Austrolimnius waterhousei* (L)	39	**4.870**	**0.037**
38. Coleoptera	Elmidae	*Coxelmis novemnotata* (L)	436	**16.126**	**0.001**
39. Coleoptera	Elmidae	*Notriolus maculatus* (L)	NEW	**11.072**	**0.003**
40. Coleoptera	Elmidae	*Notriolus* small (L)	346	**13.407**	**0.001**
41. Coleoptera	Gyrinidae	Gyrinidae (L)	50	0.381	0.543
42. Coleoptera	Hydrophilidae	*Berosus* sp. (A)	LOST	**5.207**	**0.032**
43. Coleoptera	Hydrophilidae	Hydrophilidae (L)	-52	2.406	0.134
44. Coleoptera	Scirtidae	Scirtidae (L)	34	0.357	0.556
45. Trichoptera	Calamoceratidae	*Anisocentropus* sp.	-8	0.017	0.897
46. Trichoptera	Ecnomidae	*Ecnomus continentalis*	15	0.310	0.583
47. Trichoptera	Ecnomidae	*Ecnomus* sp. small	LOST	2.314	0.141
48. Trichoptera	Hydrobiosidae	*Apsilochorema* sp. small	16	0.037	0.849
49. Trichoptera	Hydrobiosidae	Hydrobiosidae small	79	1.141	0.296
50. Trichoptera	Hydrobiosidae	*Taschorema complex* small	382	**20.437**	**<0.001**
51. Trichoptera	Hydrobiosidae	*Taschorema evansi*	53	1.545	0.226
52. Trichoptera	Hydrobiosidae	*Ulmerochorema lentum*	-7	0.025	0.876
53. Trichoptera	Hydropsychidae	*Asmicridea* sp. AV1	113	1.535	0.227
54. Trichoptera	Hydropsychidae	*Cheumatopsyche* small	256	**54.023**	**<0.001**
55. Trichoptera	Hydropsychidae	*Cheumatopsyche* sp. AV1	18	0.050	0.825
56. Trichoptera	Hydropsychidae	*Cheumatopsyche* sp. AV2	136	4.143	0.053
57. Trichoptera	Hydropsychidae	*Cheumatopsyche* sp. AV4	24	1.600	0.218
58. Trichoptera	Hydroptilidae	*Hellyethira simplex*	175	**6.666**	**0.016**
59. Trichoptera	Hydroptilidae	*Hydroptila scamandra*	169	**20.922**	**<0.001**
60. Trichoptera	Hydroptilidae	Hydroptilidae small	21	0.499	0.487
61. Trichoptera	Hydroptilidae	*Oxyethira* sp.	LOST	**8.618**	**0.007**
62. Trichoptera	Leptoceridae	*Lectrides* sp.	LOST	**7.675**	**0.011**
63. Trichoptera	Leptoceridae	Leptoceridae small	61	2.194	0.152
64. Trichoptera	Leptoceridae	*Oecetis* spp.	-40	2.225	0.149
65. Trichoptera	Leptoceridae	*Triplectides ciuskus*	-52	2.063	0.164
66. Trichoptera	Leptoceridae	*Triplectides similis*	NEW	**25.989**	**<0.001**
67. Trichoptera	Leptoceridae	*Triplectides* spp. small	LOST	3.569	0.071
68. Trichoptera	Trichoptera	Trichoptera very small	136	0.224	0.64

Appendix S1 Where does generality come from? A brief review of the literature

We conducted a brief review of the literature (50 most relevant articles) to determine whether research that tested hypotheses using multiple rivers selected study rivers according to sampling criteria that permits inference to rivers that were not part of the sample.

Web of Science (Clarivate) was used to locate 50 studies that had sampled multiple rivers to test hypotheses. An initial search used "multi rivers" or "replic rivers" across the entire database; this produced 14 studies that were relevant. A second search used "rivers" and "invertebrates" as key words, then the list was restricted to those articles in the discipline of ecology; 36 papers were chosen from a list in order of "relevance". To qualify for inclusion, studies had to be empirical studies that either collected original data or made use of available databases and tested hypotheses regarding animals of some kind (papers examining variation in hydrology, sediments, etc. but without faunal data were excluded).

Each paper was scanned to determine whether:

(1) the authors had either explicitly or implicitly described a statistical population from which rivers would be selected; this required a clear spatial boundary for the population, recognition of the number of rivers or river lengths within the study area, and explicit criteria that rivers were required to meet to qualify for selection for the study,

(2) when there were more rivers than could be sampled, whether rivers were selected randomly from all those that qualified for inclusion, and

(3) whether the authors explicitly or implicitly made inferences about other rivers from their findings

Only three of 50 papers provided a description of a clear statistical population from which rivers would be selected (Table S8). Of those three, two provided a sufficient description of the selection process to demonstrate that choices of rivers were a random selection or that all rivers that met the selection criteria were included.

Despite this, a majority of studies contained outcomes that suggested their results explicitly or implicitly applied to rivers that were not included in their sample. This finding does not imply that these papers' conclusions were necessarily unfounded; such a conclusion depends critically on the exact hypotheses that had been posed, which we did not assess. Nevertheless, most papers selected rivers without explaining why specific systems were chosen over others that may also have been suitable. Some studies also deemed individual rivers to be "representative" of rivers of a particular type, again without explaining how or why that claim could be supported.

Table S8 A list of 50 papers and whether each paper had described a formal statistical population, used a random sampling process to select rivers, and drew conclusions about rivers that were not sampled. In most cases, answers were a clear yes or no, but where there was a lack of clarity, this was noted

	Formal statistical population?	Formal sampling protocol for random choices of rivers spelled out?	Drew conclusions about rivers that were not sampled?
Allan et al. (2006)	no	no	yes
Anna et al. (2009)	no	no	yes
Blanchette & Pearson (2012)	no	no	yes
Blanchette & Pearson (2013)	no	no	yes
Bletter et al. (2015)	no	no	yes
Bright et al. (2010)	no	no	yes
Brown & Braithwaite (2005)	no	no	yes
Datry et al. (2014)	no	no	yes
Death et al. (2015)	no	no	yes
Dettmers et al. (2001)	no	no	yes
Downes et al. (2000)	yes	yes	no
Eggleton et al. (2004)	no	no	yes
Engman et al. (2017)	no	no	yes
Fell et al. (2019)	no	no	yes
Gehrke & Harris (2000)	yes	yes	yes
Gray and Harding (2009)	no	no	yes
Greenwood & Booker (2015)	no	no	yes
Greenwood & McIntosh (2010)	no	no	yes
Grumiaux & Dhainaut-Courtois (1996)	no	no	yes
Halabowski et al. (2022)	no	no	yes
Hall et al. (2006)	no	no	yes
Harrison et al. (2004)	no	Possible, but unclear	yes
Jackson et al. (2020)	no	no	yes
Jardine et al. (2012)	no	no	yes
Kietzka et al. (2019)	no	no	yes
Klose & Cooper (2012)	no	no	yes
Knight et al. (2008)	no	no	yes
Kyrlov (2002)	no	no	yes
Larsen & Ormerod (2010)	no	no	yes
Leeseburg & Keeley (2014)	no	no	no
Leigh & Datry (2017)	no	no	yes
Lusardi et al. (2016)	no	no	yes
Milner et al. (2001)	no	no	no

Monk et al. (2008)	no	no	yes
Norris et al. (2007)	yes	no	yes
Nunes et al. (2021)	no	no	yes
Patang & Soegianto (2020)	no	no	no
Petkovska & Urbanič (2015)	no	no	yes
Plichard et al. (2020)	no	no	yes, partially
Sahashi et al. (2015)	no	Partially but unclear	yes
Sanmiguel et al. (2016)	no	no	no
Spears et al. (2022)	no	no	yes
Steel et al. (2018)	no	no	yes
Stewart & Samways (1998)	no	no	yes
Strayer (1999)	no	no	no
Thompson et al. (2018)	no	no	yes
Tiunova (2006)	no	no	yes
Vinson & Dinger (2008)	no	no	yes
Wilson et al. (2021)	no	no	yes
Yubahara et al. (2015)	no	no	yes

Supplementary material References

Allan et al. (2006) Limnology of Andean piedmont rivers of Venezuela. J. N. Am. Benthol. Soc. 25: 66–81

Anna et al. (2009) Do intermittent and ephemeral Mediterranean rivers belong to the same river type? Aquat. Ecol. 43: 465–476

Blanchette & Pearson (2012) Macroinvertebrate assemblages in rivers of the Australian dry tropics are highly variable. Freshwat. Sci. 31: 865-881

Blanchette & Pearson (2013) Dynamics of habitats and macroinvertebrate assemblages in rivers of the Australian dry tropics. Freshwat. Biol. 58: 742–757

Bletter et al. (2015) The impact of significant input of fine sediment on benthic fauna at tributary junctions: a case study of the Bermejo–Paraguay River confluence, Argentina. Ecohydrology 8: 340–352

Bright et al. (2010) Variation in invertebrate and fish communities across floodplain ecotones of the Altamaha and Savannah Rivers. Wetlands 30: 1117–1128

Brown & Braithwaite (2005) Effects of predation pressure on the cognitive ability of the poeciliid *Brachyraphis episcopi*. Behav. Ecol. 16: 482–487

Datry et al. (2014) Broad-scale patterns of invertebrate richness and community composition in temporary rivers: effects of flow intermittence. Ecography 37: 94–104

Death et al. (2015) How good are Bayesian belief networks for environmental management? A test with data from an agricultural river catchment. Freshwat. Biol. 60: 2297–2309

Dettmers et al. (2001) Life in the fast lane: fish and foodweb structure in the main channel of large rivers. J. N. Am. Benthol. Soc. 20: 255–265

Downes et al. (2000) What's in a site? Variation in lotic macroinvertebrate density and diversity in a spatially replicated experiment. Austral Ecol. 25: 128–139

Eggleton et al. (2004) Assessing the potential for fish predation to impact zebra mussels (*Dreissena polymorpha*): insight from bioenergetics models. Ecol. Freshwat. Fish 13: 85–95

Engman et al. (2017) Recruitment phenology and pelagic larval duration in Caribbean amphidromous fishes. Freshwat. Sci. 36: 851–865

Fell et al. (2019) Declining glacier cover threatens the biodiversity of alpine river diatom assemblages. Glob Change Biol. 24: 5828–5840

Gehrke & Harris (2000) Large-scale patterns in species richness and composition of temperate riverine fish communities, south-eastern Australia. Mar. Freshwat. Res. 51: 165–82

Gray and Harding (2009) Braided river benthic diversity at multiple spatial scales: a hierarchical analysis of β diversity in complex floodplain systems. J. N. Am. Benthol. Soc. 28 : 537–551

Greenwood & Booker (2015) The influence of antecedent floods on aquatic invertebrate diversity, abundance and community composition. Ecohydrology 8: 188–203

Greenwood & McIntosh (2010) Low river flow alters the biomass and population structure of a riparian predatory invertebrate. Freshwat. Biol. 55: 2062–2076

Grumiaux & Dhainaut-Courtois (1996) Benthic macroinvertebrate communities of the Canal à Grand Gabarit, Aa River, and the Canalized Aa River in the North of France. J. Freshwat. Ecol. 11: 131-138

Halabowski et al. (2022) Diversity of rotifers in small rivers affected by human activity. Diversity 14: 127

Hall et al. (2006) Extremely high secondary production of introduced snails in rivers. Ecol. Appl. 16: 1121-1131

Harrison et al. (2004) The effect of instream rehabilitation structures on macroinvertebrates in lowland rivers. J. Appl. Ecol. 41: 1140-1154

Jackson et al. (2020) River food chains lead to riparian bats and birds in two mid-order rivers. Ecosphere 11:e03148

Jardine et al. (2012) Consumer-resource coupling in wet-dry tropical rivers. J. Anim. Ecol. 81: 310-322

Kietzka et al. (2019) Applying the umbrella index across aquatic insect taxon sets for freshwater assessment. Ecol. Ind. 107: 105655

Klose & Cooper (2012) Contrasting effects of an invasive crayfish (*Procambarus clarkii*) on two temperate stream communities. Freshwat. Biol. 57: 526–540

Knight et al. (2008) Relating streamflow characteristics to specialized insectivores in the Tennessee River Valley: a regional approach. Ecohydrology 1: 394–407

Kyrlov (2002) Activity of beavers as an ecological factor affecting the zooplankton of small rivers. Russ. J. Ecol. 33: 349–355

Lancaster, J., and B. J. Downes. 2017. A landscape-scale field experiment reveals the importance of dispersal in a resource-limited metacommunity. Ecology **98**:565-575.

Larsen & Ormerod (2010) Combined effects of habitat modification on trait composition and species nestedness in river invertebrates. Biol. Cons. 143: 2638–2646

Leeseburg & Keeley (2014) Prey size, prey abundance, and temperature as correlates of growth in stream populations of cutthroat trout. Environ Biol Fish 97: 599–614

Leigh & Datry (2017) Drying as a primary hydrological determinant of biodiversity in river systems: a broad-scale analysis. Ecography 40: 487–499

Lusardi et al. (2016) Environment shapes invertebrate assemblage structure differences between volcanic springfed and runoff rivers in northern California. Freshwat. Sci. 35: 1010–1022

Milner et al. (2001) Longitudinal distribution of macroinvertebrates in two glacier-fed New Zealand rivers. Freshwat. Biol. 46: 1765-1775

Motulsky, H. (1995) Intuitive Biostatistics. New York: Oxford University Press

Monk et al. (2008) Macroinvertebrate community response to inter-annual and regional river flow regime dynamics. River. Res. Applic. 24: 988–1001

Norris et al. (2007) Very-broad-scale assessment of human impacts on river condition. Freshwat. Biol. 52: 959–976

Nunes et al. (2021) Local and regional drivers of macrophyte beta diversity in tropical coastal rivers. Freshwat. Sci. 40: 138–150.

Patang & Soegianto (2020) Oxidative responses of macro-invertebrates in relation to environmental variables in rivers of East Kalimantan, Indonesia. Chem. Ecol. 36: 855-867

Petkovska & Urbanič (2015) The links between river morphological variables and benthic invertebrate assemblages: comparison among three European ecoregions. Aquat. Ecol. 49: 159–173

Plichard et al. (2020) Predictive models of fish microhabitat selection in multiple sites accounting for abundance overdispersion. River Res. Applic. 36: 1056–1075

Sahashi et al. (2015) An evaluation of the contribution of hatchery stocking on population density and biomass: a lesson from masu salmon juveniles within a Japanese river system. Fisheries Manag Ecol. 22: 371–378

Sanmiguel et al. (2016) Recovery of the algae and macroinvertebrate benthic community after *Didymosphenia geminata* mass growths in Spanish rivers. Biol Invasions 18:1467–1484

Spears et al. (2022) Assessing multiple stressor effects to inform climate change management responses in three European catchments. Inland Waters 12: 94-106

Steel et al. (2018) Associating metrics of hydrologic variability with benthic macroinvertebrate communities in regulated and unregulated snowmelt-dominated rivers. Freshwat. Biol. 63: 844–858

Stewart & Samways (1998) Conserving dragonfly (Odonata) assemblages relative to river dynamics in an African savanna game reserve. Cons. Biol. 12: 683-692

Strayer (1999) Use of flow refuges by unionid mussels in rivers. J. N. Am. Benthol. Soc. 18: 468-476

Thompson et al. (2018) Large woody debris “rewilding” rapidly restores biodiversity in riverine food webs. J Appl. Ecol. 55: 895–904

Tiunova (2006) Trophic structure of invertebrate communities in ecosystems of salmon rivers in the southern Far East. Russ. J. Ecol. 37: 419–425

Victorian Department of Environment Land Water & Planning (DELWP). 2010. Index of Stream Condition (ISC3) The Third Benchmark of Victorian River Condition. DELWP, Melbourne, Victoria.

Vinson & Dinger (2008) Aquatic invertebrates of the Grand Staircase-Escalante National Monument, Utah. Southwest. Nat. 53: 374-384

Wilson et al. (2021) Anthropogenic litter is a novel habitat for aquatic macroinvertebrates in urban rivers. Freshwat. Biol. 66: 524–534

Yubahara et al. (2015) Predicting long-term changes in riparian bird communities in floodplain landscapes. River Res. Applic. 31: 109–119
